# Lung mesenchymal stromal cells influenced by Th2 cytokines mobilize neutrophils and facilitate metastasis by producing complement C3

**DOI:** 10.1038/s41467-021-26460-z

**Published:** 2021-10-27

**Authors:** Zhiyuan Zheng, Ya-nan Li, Shanfen Jia, Mengting Zhu, Lijuan Cao, Min Tao, Jingting Jiang, Shenghua Zhan, Yongjing Chen, Ping-Jin Gao, Weiguo Hu, Ying Wang, Changshun Shao, Yufang Shi

**Affiliations:** 1 The Third Affiliated Hospital of Soochow University/The First People’s Hospital of Changzhou, State Key Laboratory of Radiation Medicine and Protection, Institutes for Translational Medicine of Soochow University, Suzhou, Jiangsu China; 2 Key Laboratory of Tumor Microenvironment and Immune Therapy of Zhejiang Province, Cancer Center, Department of Breast Surgery, The Second Affiliated Hospital, Zhejiang University, Hangzhou, Zhejiang China; 3 The First Affiliated Hospital of Soochow University/The First People’s Hospital of Suzhou, Suzhou, Jiangsu China; 4 CAS Key Laboratory of Tissue Microenvironment and Tumor, Shanghai Institute of Nutrition and Health, University of Chinese Academy of Sciences, Chinese Academy of Sciences, Shanghai, China; 5 Fudan University Shanghai Cancer Center and Institutes of Biomedical Sciences, Collaborative Innovation Center of Cancer Medicine, Shanghai Medical College, Fudan University, Shanghai, China

**Keywords:** Cancer microenvironment, Tumour immunology, Metastasis

## Abstract

Pre-metastatic niche formation is critical for the colonization of disseminated cancer cells in distant organs. Here we find that lung mesenchymal stromal cells (LMSCs) at pre-metastatic stage possess potent metastasis-promoting activity. RNA-seq reveals an upregulation of complement 3 (C3) in those LMSCs. C3 is found to promote neutrophil recruitment and the formation of neutrophil extracellular traps (NETs), which facilitate cancer cell metastasis to the lungs. C3 expression in LMSCs is induced and sustained by Th2 cytokines in a STAT6-dependent manner. LMSCs-driven lung metastasis is abolished in Th1-skewing *Stat6*-deficient mice. Blockade of IL-4 by antibody also attenuates LMSCs-driven cancer metastasis to the lungs. Consistently, metastasis is greatly enhanced in Th2-skewing *T-bet*-deficient mice or in nude mice adoptively transferred with *T-bet*-deficient T cells. Increased C3 levels are also detected in breast cancer patients. Our results suggest that targeting the Th2-STAT6-C3-NETs cascade may reduce breast cancer metastasis to the lungs.

## Abstract (web summary)

The formation of the pre-metastatic niche enables the colonisation of disseminated cancer cells in distant organs. Here, the authors show that Th2 cytokines induce Complement 3 production in lung mesenchymal stromal cells, which recruits neutrophils and promotes the formation neutrophil extracellular traps, facilitating breast cancer cell metastasis to the lungs.

## Introduction

Metastasis, the major cause of cancer-related death, consists of dissemination and secondary colonization of cancer cells^1^. Disseminated tumor cells face many obstacles and only few of them can colonize in distant organs. Therefore, colonization in distant organs is a rate-limiting step of metastasis^2,3^. The local microenvironment in the distant organ determines whether or not colonization of cancer cells can establish^4–6^. Primary tumors are known to facilitate the formation of a favorable microenvironment at the secondary sites, also known as pre-metastatic (PM) niche^7–9^. Tumor-derived growth factors^10^, inflammatory cytokines^11^, chemokines^12^, and exosomes^13,14^ are known to be critical for the initiation and evolution of PM niche. Immune cells, such as neutrophils^15,16^, macrophages^17,18^, myeloid-derived suppressor cells^12,19^, and T cells^20,21^, are believed to be responders to the tumor-secreted factors and represent critical constituents of PM niche. Neutrophils, e.g., were shown to secret leukotrienes that select for metastasis-initiating cancer cells^15^. Neutrophil extracellular traps (NETs) formed by activated neutrophils were recently demonstrated to remodel extracellular matrix to awaken dormant cancer cells and enable their metastasis^22^.

In addition to cells from the hematopoietic lineage, local stromal and epithelial cells also contribute to PM niche. It has been reported that VEGFR1^+^ hematopoietic bone marrow progenitors expressing VLA-4 interact with fibronectin derived from resident fibroblasts to initiate the formation of PM niche^8^. Lung fibroblasts and epithelial cells can uptake tumor-derived exosomes and govern the lung tropism^4,14^. Mesenchymal stromal cells (MSCs) exist in almost every type of tissues and are critical for maintaining tissue homeostasis and regeneration^23^. During tumor development, bone marrow and tissue-resident MSCs can be mobilized to modulate tumor cells directly or indirectly. Various growth factors and chemokines can be produced by tumor MSCs, especially upon interaction with immune cells and inflammatory factors, to influence tumor growth and metastasis. Also, MSCs associated with primary tumors contribute to tumor progression by orchestrating tumor immune microenvironment, including recruitment of monocytes, macrophages, and neutrophils, and enhancement of tumor-promoting immune responses^24–26^. However, our knowledge of how local MSCs contribute to the PM niche and how they interact with immune cells in forming the PM niche is very limited.

In this work, we demonstrate that resident MSCs are critically involved in the formation of pulmonary PM niche of breast cancer cells. MSCs are found to recruit neutrophils to the lung and convert them into NETs by producing complement component 3 (C3). The formation of NETs is required for the pro-metastatic effect of MSCs. We further show that the upregulation of C3 in the pulmonary PM niche during tumor development is driven by Th2 cytokines through the STAT6 signaling pathway. Thus, this study reveals how local MSCs respond to inflammatory cytokines and how they prepare the PM niche for the colonization of cancer cells in the lungs.

## Results

### Distant PM MSCs acquire metastasis-promoting property

**Fig. 1:**
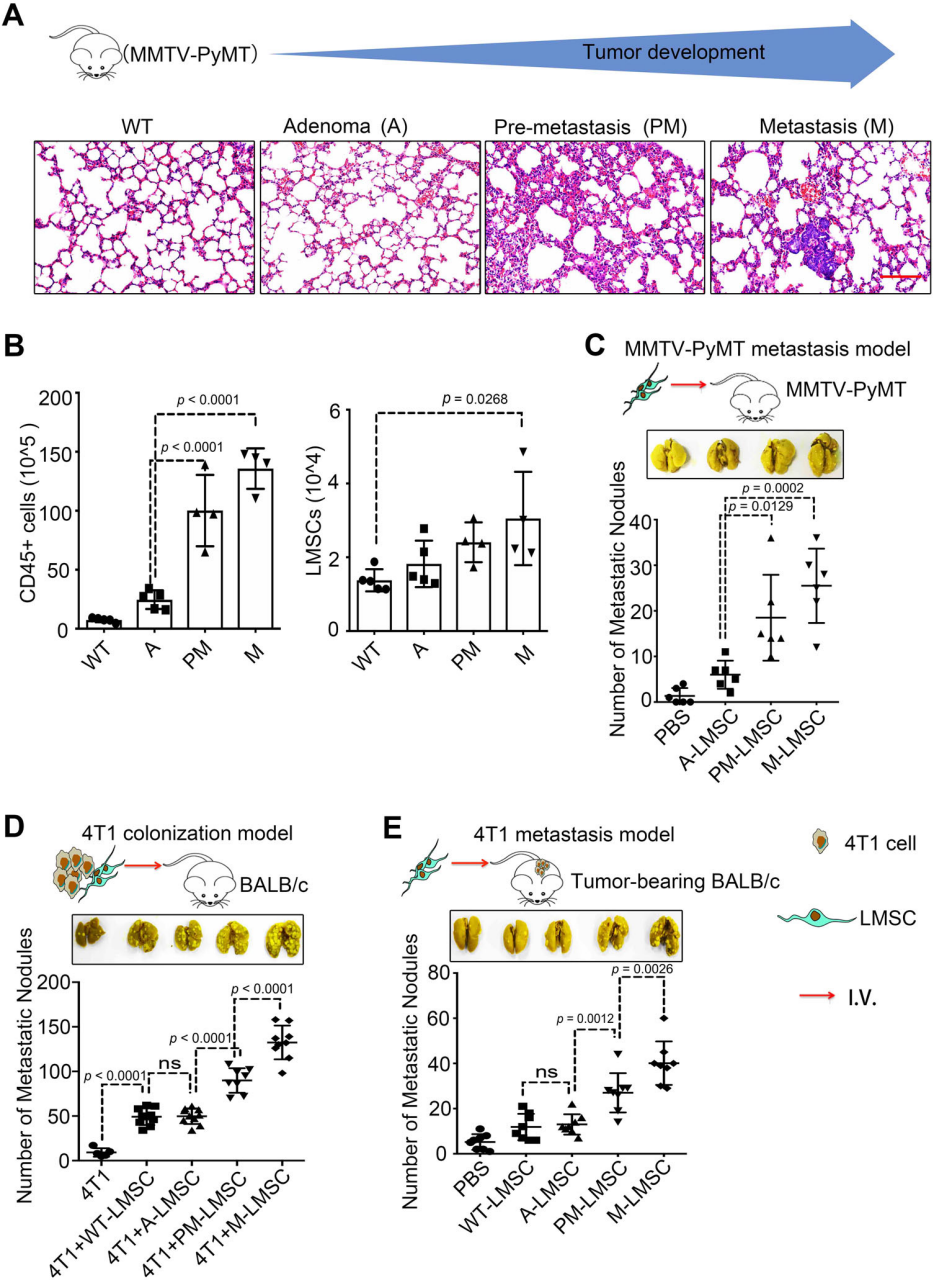
LMSCs acquire increased metastasis-promoting ability along with tumor progression. **A** H&E staining of lung sections from MMTV-PyMT mice at different tumor stages, adenoma (A), pre-metastatic (PM), and metastatic (M) stage. Scale bar represents 100 μm. The images were representative of those generated from five mice in each group. **B** Single-cell suspensions prepared from lung tissues of MMTV-PyMT mice at various time points were enumerated and analyzed by flow cytometry for CD45^+^ immune cells and 7AAD^−^ Lineage^−^ Sca1^+^ CD44^+^ LMSCs. *n* = 5, 5, 4, and 4 mice. **C** PM- or M-LMSCs promote lung metastasis in MMTV-PyMT mice. PBS or A-, PM-, and M-LMSCs (1 × 10^5^) were administered into MMTV-PyMT mice at the adenoma stage. Five weeks later, lung metastatic nodules (LMNs) were counted. *n* = 6 mice per group. **D** PM- or M-LMSCs promote lung colonization of intravenously injected 4T1 breast cancer cells. 4T1 cells were co-administered with WT-LMSCs from FVB or A-, PM-, and M-LMSCs from MMTV-PyMT mice at a 1 : 5 ratio (1 × 10^4^ LMSCs and 5 × 10^4^ 4T1 cells) into BALB/c mice by intravenous injection. After 14 days, LMNs were counted. From left to right: *n* = 5, 10, 10, 8, and 9 mice. **E** PM- or M-LMSCs promote lung metastasis of 4T1 cells implanted in fat pad. 4T1 cells (4 × 10^5^) were implanted into fat pads of BALB/c mice. After 10 days, WT-LMSCs or A-, PM-, and M-LMSCs (1 × 10^5^) were injected into these tumor-bearing mice by intravenous injection, respectively. LMNs were counted on day 30. *n* = 8 mice per group. All the data are presented as mean values ± SD. ns, not significant; *p* < 0.05, significant, using a one-way ANOVA with Sidak’s post test. Source data are provided as a Source Data file for Fig. 1B–E.

PM niche are distant tissue microenvironments that can be actively modified so that the tumor cells from their primary site can migrate to, settle in, and colonize at^9^. Several studies reported that neutrophils and macrophages contribute to tumor metastasis^27–29^. We set out to study the components of pulmonary PM niche and their respective roles in metastasis using the lung metastatic Mouse mammary tumor virus (MMTV)-polyoma middle T antigen (PyMT) mammary tumor mouse model^30,31^. The tumor progression in this mouse model can be divided into three stages: adenoma (A), PM and metastatic (M) stages (Fig. 1A). We were most interested in changes in the lungs at the PM stage. Histological examination of the lungs at the PM stage revealed increased thickening of alveolar walls, dilation of capillaries, and interstitial edema. Flow cytometry analysis showed increasing infiltration of CD45^+^ cells during tumor development (Fig. 1B) and the numbers of CD3^+^ T cells, neutrophils, and macrophages at PM were all markedly higher than those at the A stage (Supplementary Fig. 1A), suggesting that those immune cells are potentially involved in the formation of PM niche. We also characterized the lung MSCs (LMSCs), which were identified as Lineage^−^CD44^+^Sca1^+^ (Supplementary Fig. 1B), and found that the number of LMSCs was also increased along with tumor progression (Supplementary Fig. 1B). The concomitant expansion of immune cells and LMSCs suggest that they may interact during the formation of PM niche*.* We then obtained LMSCs from the lungs of MMTV-PyMT mice at different stages of the breast cancer development, designated as A-LMSCs, PM-LMSCs, and M-LMSCs (Supplementary Fig. 1C). Wild-type (WT) LMSCs were isolated from FVB mice (Supplementary Fig. 1C). All LMSCs were expanded in vitro for three passages for further phenotypic and functional characterization. They showed spindle-like morphology (Supplementary Fig. 1C), displayed the same pattern in surface markers, CD29^+^CD44^+^CD140a^low^Nestin^+^Sca1^low^Lineage^−^CD11b^−^CD31^−^CD34^−^CD45^−^ (Supplementary Fig. 1D). Although Sca1 staining was low, only one peak was detected, indicating that these cells may belong to one population. WT-LMSCs isolated from tumor-bearing mice were also Sca1^low^. These phenotypic features suggest that the LMSC preparations derived from MMTV-PyMT mice were largely, if not completely, free of breast cancer cells or lung epithelial cells. The LMSCs were multipotent, as demonstrated by their ability to differentiate into adipocytes and osteoblasts under established experimental conditions (Supplementary Fig. 1E), and had similar proliferation capacity (Supplementary Fig. 1F).

To determine whether LMSCs could promote tumor metastasis, we intravenously (i.v.) injected different lines of LMSCs that had been expanded in vitro into MMTV-PyMT mice at the A stage and scored metastatic nodules in the lung. PM-LMSCs or M-LMSCs greatly increased lung metastasis when compared with the A-LMSCs (Fig. 1C). To verify the finding from a different angle, we next tested the effect of LMSCs on the colonization of injected 4T1 cells (a murine breast cancer cell line). In this experimental system, 4T1 cells were of BALB/c origin and in vivo experiments involving this breast cancer cell line were all carried out in BALB/c mice. It has been reported that syngeneic, allogeneic, and even xenogeneic MSCs are all effective in modulating immune responses in vivo^23^, due to their low major histocompatibility complex (MHC) expression and strong immunosuppressive ability. We reasoned that MSCs originated from MMTV-PyMT mice of the FVB background should persist long enough in BALB/c mice to influence the lung tissue microenvironment, to allow the colonialization of metastatic cancer cells, although in an allogenic setting. Indeed, the same PM-LMSCs or M-LMSCs from the FVB background also strikingly enhanced lung colonization of i.v. co-injected 4T1 cells in BALB/c mice, when compared to WT-LMSCs or A-LMSCs (Fig. 1D). Furthermore, injection of PM-LMSCs or M-LMSCs greatly increased the number of lung metastatic nodules in mice with 4T1 cells implanted into the mammary gland fat pad (Fig. 1E). These results with 4T1 cells substantiated the proposition that PM-LMSCs and M-LMSCs possess potent metastasis-promoting property as observed in MMTV-PyMT mice.

### C3 is highly expressed in MSCs at PM niche and is required for the metastasis-promoting property of LMSCs

**Fig. 2:**
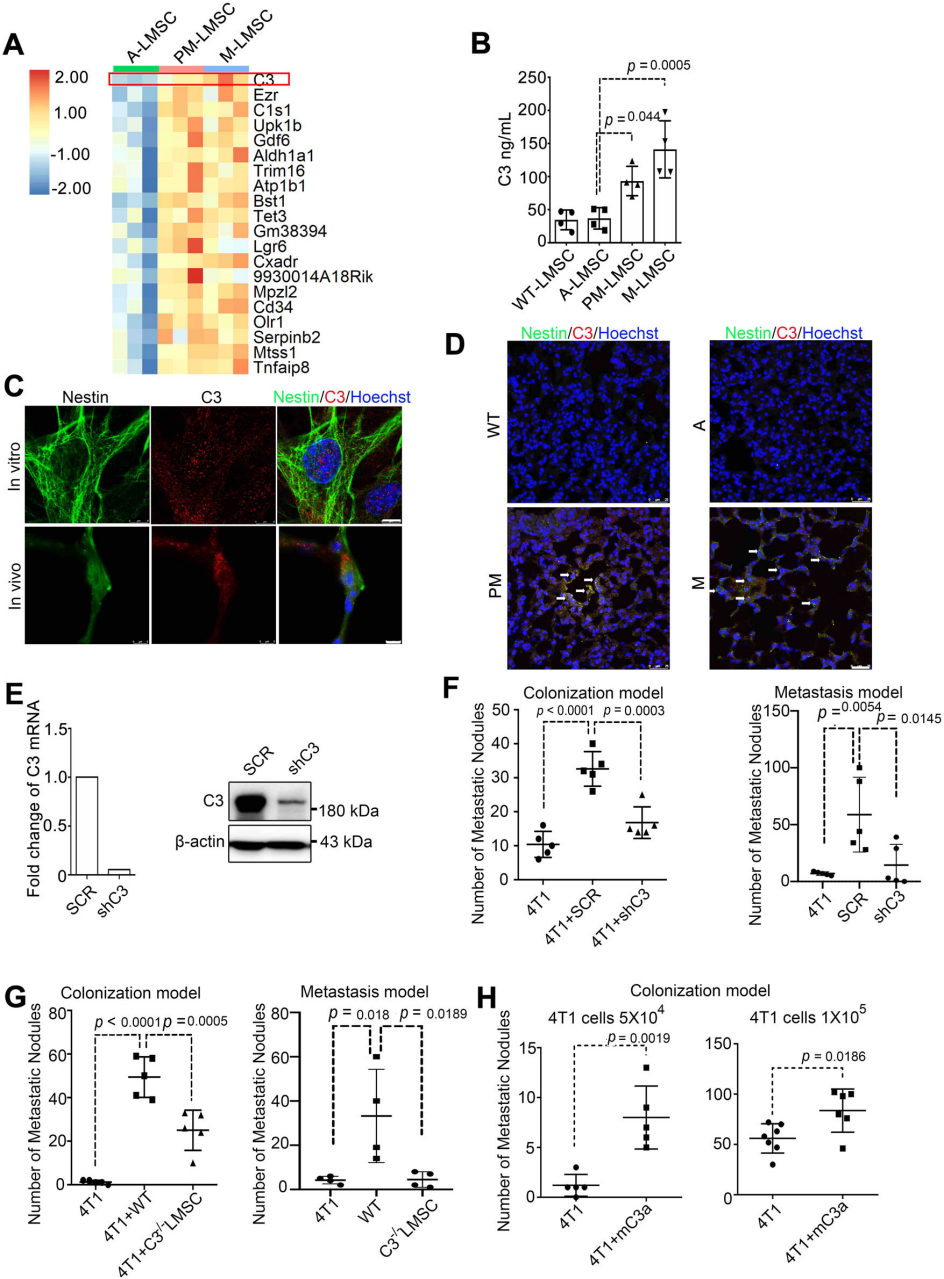
Complement component 3 is upregulated in PM-LMSCs and M-LMSCs, and mediates the metastasis-promoting effect. **A** Heatmap of the top 20 mRNAs enriched in PM-LMSCs and M-LMSCs. *n* = 3 mice each. **B** C3 levels in the supernatants of cultured WT-LMSCs, A-LMSCs, PM-LMSCs, and M-LMSCs. Fifty thousand WT, A-, PM- or M-LMSCs were grown in 12-well plates for 24 h before supernatants were collected. *n* = 4 supernatants of independently cultured LMSCs. **C** Immunofluorescence of C3 and Nestin. PM-LMSCs were co-stained for C3 (red) and Nestin (green), and lung tissue of 10- to 11-week-old MMTV-PyMT mouse was co-stained with C3 (red) and Nestin (green). Scale bar (in vitro), 8 μm; scale bar (in vivo), 5 μm. The images were representative of three independent experiments. **D** Representative images of lung of MMTV-PyMT mice stained by immunofluorescence for C3 (red) and Nestin (green). Scale bar represents 25 μm. The images were representative of those generated from three mice each group. **E** C3 was depleted by RNAi. The RNAi efficiency was shown on the left. The WB were representative of two independent experiments. **F** Depletion of C3 blocked the metastasis-promoting activity of LMSCs. SCR or shC3 PM-LMSCs were co-administered with 4T1 cells into BALB/c mice and LMNs were counted on day 14. *n* = 5 mice each (Colonization model). 4T1 cells (4 × 10^5^) were implanted into mammary gland fat pads of BALB/c mice. After 10 days, control PM-LMSCs or shC3 PM-LMSCs (1 × 10^5^) were administered into these tumor-bearing mice by i.v. The lung metastatic nodules were counted on day 30. *n* = 5 mice each (Metastasis model). **G** C3 deletion in LMSCs reduced lung colonization. WT or *C3*^−/−^ LMSCs were co-injected with 4T1 cells into BALB/c mice and LMNs were counted on day 14. *n* = 5 mice each (Colonization model). 4T1 cells (4 × 10^5^) were implanted into mammary gland fat pads. After 10 days, WT or *C3*^−/−^ LMSCs (1 × 10^5^) were administered into these tumor-bearing mice by i.v. The LMNs were counted on day 30. *n* = 4 mice each (Metastasis model). **H** Recombinant C3a (mC3a) enhanced 4T1 metastasis. mC3a was administered i.v. into 4T1 tumor-bearing mice on days 1 and 7, and LMNs were counted on day 14. *n* = 5 mice each (left), *n* = 7, 6 mice (right). All the data are presented as mean values ± SD. ns, not significant; *p* < 0.05, significant, using a one-way ANOVA with Sidak’s post test for Fig. 2B, F, G; using an unpaired, two-tailed, Student’s *t*-test for 2H. Source data are provided as a Source Data file for Fig. 2B, E–H.

To molecularly characterize the mechanisms that underlie the differential metastasis-promoting effects of the LMSCs in MMTV-pyMT mice, we analyzed the gene expression profiles in LMSCs by RNA-sequencing (RNA-seq) (Supplementary Fig. 2A). Top 20 genes that were upregulated in PM- and M-LMSCs, shown in Fig. 2A, included complement C3, a central protein in the complement system. The elevation in the expression of C3 was confirmed using quantiative PCR (Supplementary Fig. 2B). The C3 upregulation in PM- and M-LMSCs in comparison to WT- and A-LMSCs was further confirmed at the protein level, as demonstrated by enzyme-linked immunosorbent assay (ELISA) (Fig. 2B) and immunoblot analysis (Supplementary Fig. 2C). To further validate the upregulation of C3 in PM-LMSCs, we performed immunofluorescence staining with antibodies against Nestin, a marker of LMSCs^32^, and C3. Nestin and C3 signals were shown to colocalize both in cultured PM-LMSCs and in lung tissues (Fig. 2C). Furthermore, C3 was upregulated in PM and metastatic lung, and was primarily localized in Nestin-positive cells (Fig. 2D). Thus, C3 is upregulated in PM- and M-LMSCs.

To investigate whether C3 mediated the pro-metastatic effect of PM-LMSCs, we established shC3 PM-LMSC cell lines, in which C3 was depleted by RNA interference (RNAi) (Fig. 2E). When co-injected together with 4T1 cells, shC3 PM-LMSCs were far less effective in promoting lung colonization than the scramble (SCR) short hairpin RNA (shRNA)-transfected PM-LMSCs (Fig. 2F). Meanwhile, LMSC-driven lung metastasis was significantly lower when shC3 PM-LMSCs were used (Fig. 2F). Similarly, in comparison to LMSCs from WT mice, LMSCs from C3 knockout mice^33^ were greatly compromised in their ability to promote metastasis in both lung colonization and metastasis models (Fig. 2G). Moreover, i.v. injection of C3a recombinant protein to 4T1-bearing mice significantly increased the number of lung metastatic nodules (Fig. 2H). Together, these results suggest that C3 is critically required for the promotion of lung metastasis of breast cancer cells by LMSCs.

### Neutrophil recruitment to the lungs is dependent on C3a

**Fig. 3:**
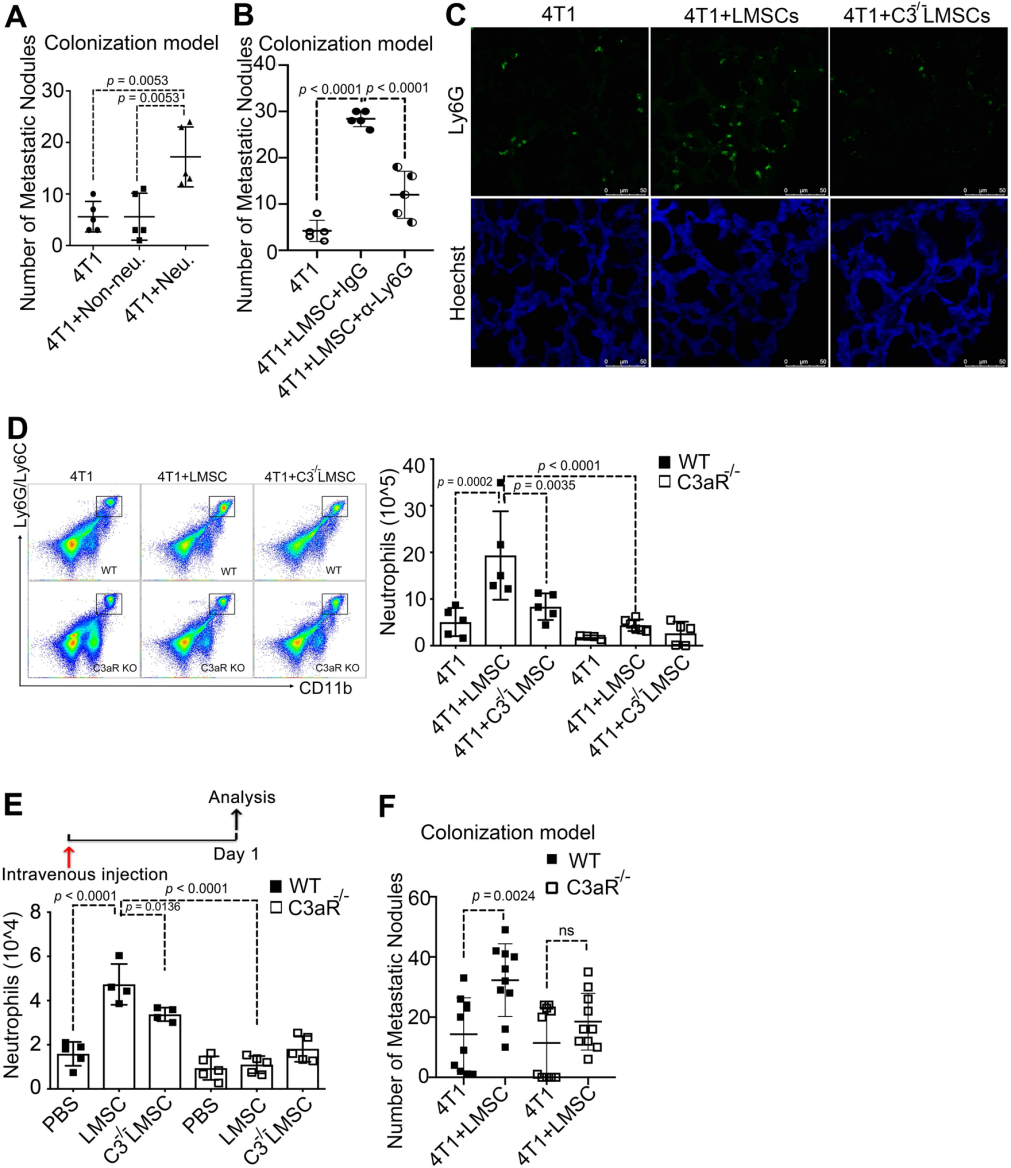
Pro-metastatic neutrophil accumulation in the pre-metastatic niche is C3a receptor dependent. **A** Adoptively transferred neutrophils promoted 4T1 tumor metastasis. Neutrophils (2 × 10^6^) and non-neutrophils (2 × 10^6^) isolated from the bone marrow of BALB/c mice via magnetic beads sorting were respectively co-administered with 4T1 cells into BALB/c mice. LMNs were counted on day 14. *n* = 5 mice each. **B** Depletion of neutrophils completely abolished the metastasis-promoting activity of LMSCs. After co-administration of LMSCs with 4T1 cells into BALB/c mice, anti-Ly6G (α-ly6G, 50 μg per mouse) or IgG was administered intraperitoneally on days 2, 4, 6, 8, 10, and 12. LMNs were counted on day 14. *n* = 5 mice each. **C** Lack of C3 eliminated lung infiltration of neutrophils. WT-LMSCs and *C3*^−/−^ LMSCs were respectively co-injected with 4T1 cells into wild-type mice and the lung tissues were stained using anti-Ly6G. Scale bar represents 50 μm. The images were representative of those generated from three mice each group. **D** C3aR expression on neutrophils was required for their accumulation in the lung. WT-LMSCs and *C3*^−/−^ LMSCs were respectively co-injected with 4T1 cells into wild-type or *C3aR*^−/−^ mice, and the population of neutrophils were analyzed on day 14 by flow cytometry. *n* = 5, 5, 5, 4, 6, and 5 mice. **E** LMSCs recruited neutrophils via the C3–C3aR axis. LMSCs or *C3*^−/−^ LMSCs injected into wild-type or *C3aR*^−/−^ mice, respectively. The mice were killed for the examination of neutrophils in the lung 24 h after injection of LMSCs. *n* = 5, 4, 4, 5, 5, and 5 mice. **F** LMSCs failed to promote metastasis in C3aR-deficient mice. LMSCs were co-injected with 4T1 cells into wild-type or *C3aR*^−/−^ mice and LMNs were counted on day 14. *n* = 10 mice each. All the data are presented as mean values ± SD. ns, not significant; *p* < 0.05, significant, using a one-way ANOVA with Sidak’s post test. Source data are provided as a Source Data file for Fig. 3A, B, D–F.

In the MMTV-PyMT model, the infiltration of neutrophils and macrophages in the lungs increased along with tumor progression (Supplementary Fig. 1A). We therefore tested whether these immune cells mediated the metastasis-promoting effect of LMSCs. Only neutrophils, but not other cells of the myeloid lineage, were found to promote breast cancer cell metastasis to the lungs when co-injected with 4T1 cells i.v. (Fig. 3A). In addition, administration of anti-Ly6G, which depletes neutrophils (Supplementary Fig. 3A), greatly impaired the LMSC-aided tumor metastasis (Fig. 3B). We reasoned that C3, produced by LMSCs, might promote the formation of PM niche by acting on neutrophils. Indeed, although neutrophil infiltration in the lungs was significantly increased when 4T1 and LMSCs were co-inoculated, depletion of C3 in LMSCs abolished the effect, as shown by immunofluorescence staining and flow cytometric analysis (Supplementary Fig. 3B). Moreover, neutrophil accumulation in the lungs was reduced when 4T1 cells were injected together with *C3*^*−/−*^ LMSCs, as compared to WT-LMSCs (Fig. 3C). Furthermore, LMSC-induced neutrophil accumulation was totally abolished in *C3aR*^*−/−*^ mice^34^ (Fig. 3D). We verified that the neutrophils highly expressed C3aR, both at the mRNA (Supplementary Fig. 3C) and at the protein level (Supplementary Fig. 3D). Similarly, when LMSCs or *C3*^*−/−*^ LMSCs alone were injected into WT or *C3aR*^*−/−*^ mice i.v., neutrophil accumulation in the lungs depended on the presence of C3 in LMSCs or the C3 receptor in the neutrophils (Fig. 3E). Intravenously injected mouse recombinant complement component C3a (mC3a) could also increase neutrophil infiltration in the lungs (Supplementary Fig. 3E). Moreover, a transwell neutrophil migration assay showed that although LMSCs (placed in the lower compartment) were highly effective in recruiting neutrophils (in the upper compartment) (Supplementary Fig. 3F), neutrophil recruitment was greatly reduced when C3 was depleted (Supplementary Fig. 3G). Conversely, neutrophil recruitment was increased by mC3a (Supplementary Fig. 3H). These results suggest that the recruitment of neutrophils by LMSCs was dependent on the C3–C3a receptor axis both in vivo and in vitro. To verify that this axis mediates lung metastasis, we i.v. injected 4T1 cells together with or without LMSCs into WT and *C3aR*^*−/−*^ mice, respectively. Indeed, LMSCs were unable to promote lung colonization in *C3aR*^−/−^ mice (Fig. 3F). Importantly, LMSCs failed to increase lung metastasis in *C3aR*^−/−^ mice (Supplementary Fig. 3I). Together, these findings suggest that LMSCs promote tumor metastasis via the MSCs (C3)–neutrophil (C3a receptor) axis.

### C3 promotes NET-mediated lung metastasis

**Fig. 4:**
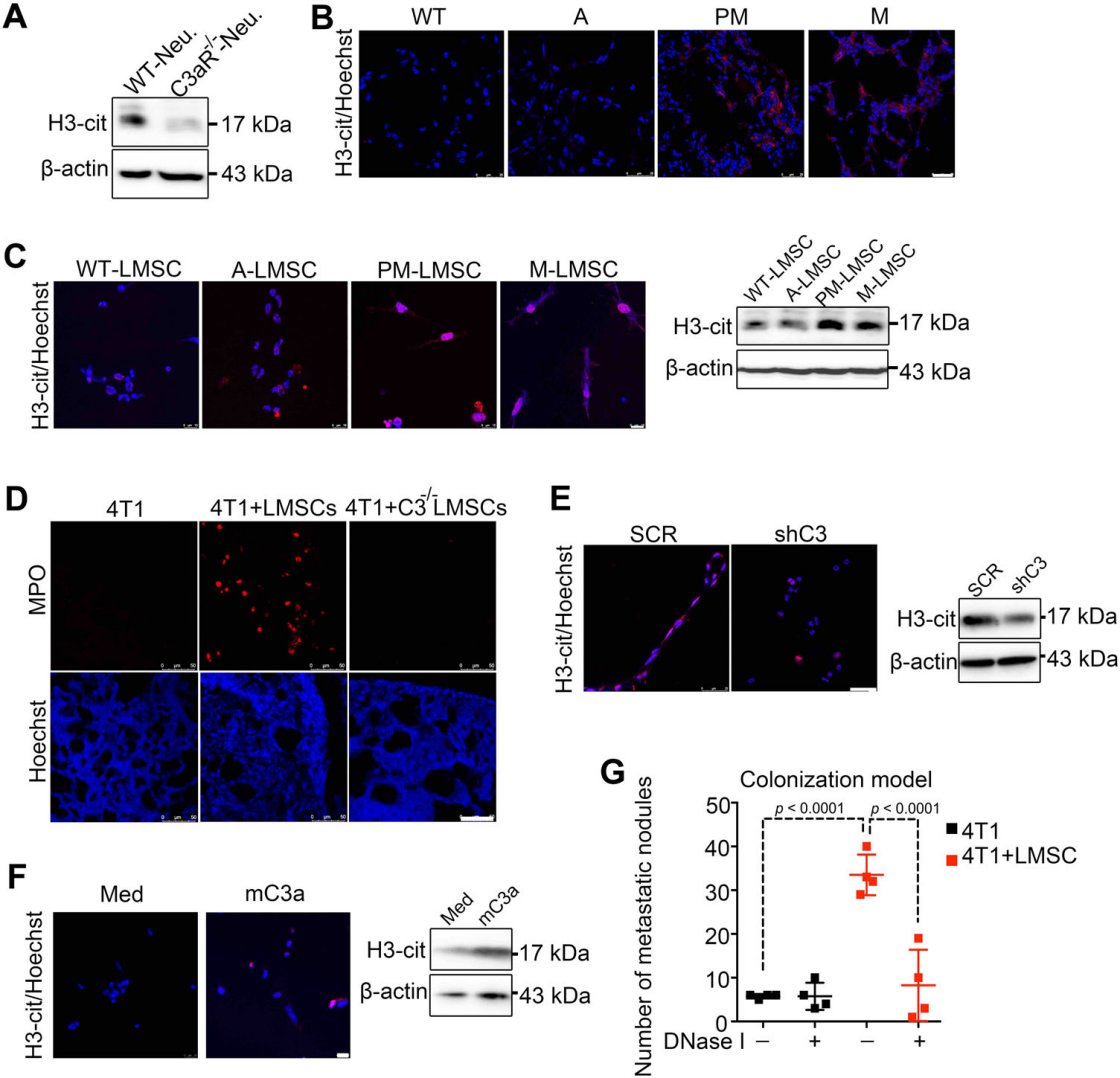
C3 promotes NET-mediated metastasis. **A** H3-cit, a marker for NETs, was reduced in *C3aR*^*−/−*^ neutrophils. Proteins from wild-type or *C3aR*^*−/−*^ neutrophils were detected using western blotting analysis. The WB images were representative of three independent experiments. **B** Representative immunofluorescence images showing the H3-cit staining (red) of lung tissues from WT-lung of FVB mice or A, PM, and metastatic lung of MMTV-PyMT mice. Scale bar represents 25 μm. The images were representative of those generated from three mice each group. **C** PM-LMSCs and M-LMSCs induced neutrophils to form NETs. Neutrophils (1 × 10^6^) were co-cultured with LMSCs from MMTV-PyMT mice at different tumor stages for 24 h and were examined for H3-cit. Left, representative images of immunofluorescence staining. Scale bar represents 10 μm. Right, increased H3-cit revealed by western blotting analysis. The IF images were representative of those generated from three independent experiments. WB was done twice. **D** NET formation in the lungs of tumor-bearing mice required C3. 4T1 cells were co-injected with WT or *C3*^*−/−*^ LMSCs into BALB/c mice. Lung tissue sections were stained anti-MPO. Scale bar represents 50 μm. The images were representative of those generated from three mice each group. **E** Knockdown of C3 in LMSCs reduced NETs in co-cultured neutrophils. Neutrophils (1 × 10^6^) were co-cultured with SCR or shC3 PM-LMSCs for 24 h and were examined for H3-cit. Scale bar represents 25 μm. Right, decreased H3-cit expression revealed by western blotting analysis. The images were representative of three independent experiments. WB was repeated once. **F** mC3a induced NET formation. Neutrophils (1 × 10^6^) were treated with recombinant mC3a (1 μg/ml) for 24 h and examined for H3-cit. Scale bar represents 10 μm. Med was the cultured medium. The images were representative of three independent experiments. WB was done twice. **G** DNase I abolished the metastasis-promoting activity of LMSCs. After LMSCs and 4T1 cells were co-injected into BALB/c mice, DNase I (400 U per mouse) was administered i.v. on days 1, 4, 7, and 10, and lung metastatic nodules were counted on day 14. *n* = 4 mice each. Data are presented as mean values ± SD. ns, not significant; *p* < 0.05, significant, using a one-way ANOVA with Sidak’s post test. Source data are provided as a Source Data file for Fig. 4A, C, E–G.

We found that *C3aR*^−/−^ neutrophils adoptively transferred i.v. into WT mice were less effective in promoting tumor metastasis than WT neutrophils (Supplementary Fig. 4A). Considering that although both WT neutrophils and *C3aR*^−/−^ neutrophils could easily reach lung upon i.v. injection but still displayed differential effects on tumor metastasis, we speculated that C3a receptor may not only facilitate neutrophil recruitment but also act by other mechanisms to promote metastasis. It is known that beside engaging in phagocytosis and degranulation of cytotoxic enzymes, neutrophils can also kill microorganisms through forming NETs, which contain cytotoxic enzymes, proteases, and chromatin DNA, and are released into the extracellular space^35,36^. NETs do not only function to combat infections but also play a role in noninfectious inflammatory diseases^37–40^. NET formation in response to bacterial infection or tobacco smoke exposure was shown to promote re-colonization of cancer cells^22^. Interestingly, *C3aR*^*−/−*^ neutrophils hardly form NETs^41^. We observed that, compared with WT neutrophils, *C3aR*^*−/−*^ neutrophils hardly expressed H3-cit, a chromatin marker for NETs (Fig. 4A). Furthermore, we examined the levels of H3-cit in the MMTV-PyMT model during the course of tumor development. Immunofluorescence staining and immunoblot analysis showed that the level of H3-cit was higher in PM and M lungs than in normal and A-stage lungs (Fig. 4B and Supplementary Fig. 4B). Co-culture of neutrophils and LMSCs from mice at different tumor stages showed that neutrophils exposed to PM- or M-LMSCs displayed a higher level of H3-cit (Fig. 4C). We also found that Pad4 and myeloperxodase (Mpo), which are associated with NETs^42^, were all upregulated in neutrophils treated with PM-LMSCs in comparison to those treated with A-LMSCs (Supplementary Fig. 4C). We next tested whether the increased NETs caused by PM-LMSCs was mediated by C3. Indeed, Mpo was nearly absent in the *C3*^*−/−*^ LMSC-treated group (Fig. 4D). Furthermore, the H3-cit level was significantly reduced in neutrophils co-cultured with shC3 LMSCs (Fig. 4E). In addition, NET-associated genes were also downregulated (Supplementary Fig. 4D). Moreover, C3a recombinant protein increased the H3-cit level, both in vitro and in vivo (Fig. 4F and Supplementary Fig. 4F), and upregulated NET-associated genes in neutrophils (Supplementary Fig. 4E). These results suggest that the C3–C3aR axis is important for NETs formation. Importantly, when DNase I, which destroys NETs, was administered i.v., the LMSC-conferred metastasis was abolished (Fig. 4G). Together, these findings strongly argue that the pro-metastatic effect of C3 is mediated by the formation of NETs via the C3–C3a receptor axis.

### IL-4/IL-13-STAT6 signaling induces C3 expression in LMSCs

**Fig. 5:**
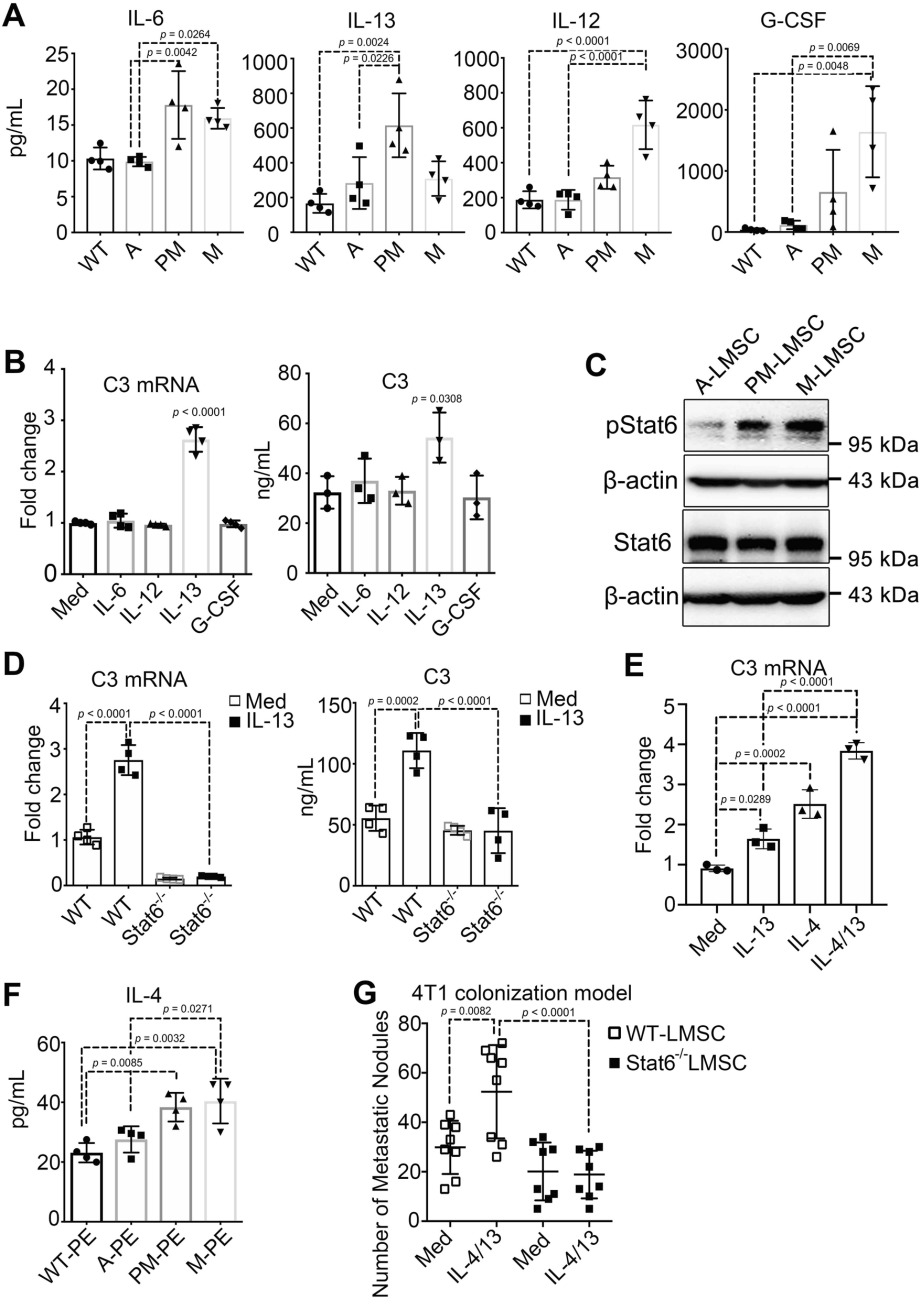
C3 expression is upregulated by the IL-4/IL-13-STAT6 signaling pathway. **A** Levels of IL-6, IL-13, IL-12, and G-CSF were increased in serum of mice with advanced tumor stages. Serum derived from MMTV-PyMT at different time points were measured via 23 cytokines multiplexed bead immunoassay. *n* = 4 mice each. **B** C3 expression in LMSCs was induced by IL-13. LMSCs in culture were treated with IL-6, IL-13, IL-12, or G-CSF (10 ng/ml for 24 hr, respectively), and mRNA and supernatant from the treated LMSCs were examined for C3. Med represents cultured medium. *n* = 4 (left panel), *n* = 3 (right panel) individual experiments. **C** Levels of phosphorylated STAT6 and total STAT6 in LMSCs. Proteins from 5 × 10^4^ A-, PM-, or M-LMSCs grown in 12-well plates for 24 h were collected and detected through immunoblotting. The WB images were representative of two independent experiments. **D** STAT6 is required for IL-13-induced C3 expression. mRNA and supernatant from cultured WT or *Stat6*^*−/−*^ LMSCs treated with or without IL-13 (10 ng/ml for 24 h) were examined for C3. Med represents cultured medium. *n* = 4 individual experiments. **E** IL-4 and IL-13 potently induced C3. C3 mRNA from cultured LMSCs treated with IL-4, IL-13, or IL-4/IL-13 (10 ng/ml for 24 h) was analyzed by qRT-PCR. Med represent cultured medium. *n* = 3 individual experiments. **F** Increasing IL-4 expression with advancing tumor development. IL-4 in pulmonary extracts (PEs) derived from MMTV-PyMT mice at different tumor stages were measured by ELISA. *n* = 4 mice each. **G** *Stat6*-deficient LMSCs did not acquire IL-4/IL-13-conferred metastasis-promoting ability. WT or *Stat6*^*−/−*^ LMSCs treated with or without IL-4/IL-13 (20 ng/ml) were co-administered with 4T1 cells into BALB/c mice and lung metastatic nodules were counted on day 14. Med was the cultured medium. *n* = 8 mice each. All the data are presented as mean values ± SD. ns, not significant; *p* < 0.05, significant, using a one-way ANOVA with Sidak’s post test. Source data are provided as a Source Data file.

As innate and adaptive immune cells accumulate in the PM niche (Supplementary Fig. 1A), they could interact with and reprogram the function of LMSCs. Alternatively, primary tumors could produce systemic factors that directly contribute to the formation of PM niche. To determine what is responsible for the C3 increase in PM-LMSCs, we examined the levels of 23 inflammatory factors in serum of MMTV-PyMT mice at different stages of tumor development using the Luminex microbead-based multiplex cytokine array assay. Four inflammatory factors, interleukin (IL)-6, IL-13, IL-12, and granulocyte colony-stimulating factor (G-CSF), were found to be significantly upregulated at PM and M stages (Fig. 5A). We treated A-LMSCs with each of the four cytokines, respectively, and found that only IL-13 could induce C3 expression (Fig. 5B). STAT6 plays a central role in mediating the biological effects of IL-4/IL-13. Upon phosphorylation by receptor-associated kinases, STAT6 forms a homodimer and is then translocated to the nucleus to act as a transcription factor^43^. We observed that the phosphorylation levels of STAT6 in PM-LMSCs and M-LMSCs were markedly higher than that in A-LMSCs (Fig. 5C). To determine whether STAT6 mediates the increased C3 expression caused by IL-13, we depleted Stat6 in LMSCs by RNAi. Depletion of Stat6 abolished IL-13-induced C3 expression (Supplementary Fig. 5A). Moreover, the basal and IL-13-induced C3 expression levels were greatly reduced in *Stat6*^−/^^−^ LMSCs when compared with WT-LMSCs (Fig. 5D). As IL-13 is closely related to and has overlapping functions with IL-4, through the classical STAT6 signaling pathway, we applied IL-13 and IL-4, each alone and in combination, to LMSCs. As expected, treatment with both cytokines resulted in a higher expression level of C3 than IL-13 treatment alone (Fig. 5E). It should be emphasized that although IL-4 was undetectable in the serum, its expression in pulmonary extract (PE) was found to be higher in PM and M stages (Fig. 5F), indicating that IL-4 could be released locally in the lungs. Therefore, IL-4/IL-13-STAT6 signaling probably mediated the C3 upregulation. Importantly, only WT-LMSCs, but not *Stat6*^−/−^ LMSCs, acquired increased metastasis-promoting property when treated with IL-4/IL-13 (Fig. 5G). Furthermore, immunofluorescence assay showed that C3 (green) and H3-cit (red) levels were upregulated only by the WT-LMSCs, but not by the Stat6^−/−^ LMSCs, when treated with IL-4/IL-13 (Supplementary Fig. 5B). These results thus strongly suggest that IL-4/IL-13 can induce C3 expression via the STAT6 signaling pathway.

### LMSCs acquire a potent pro-metastatic property under Th2 inflammation

**Fig. 6:**
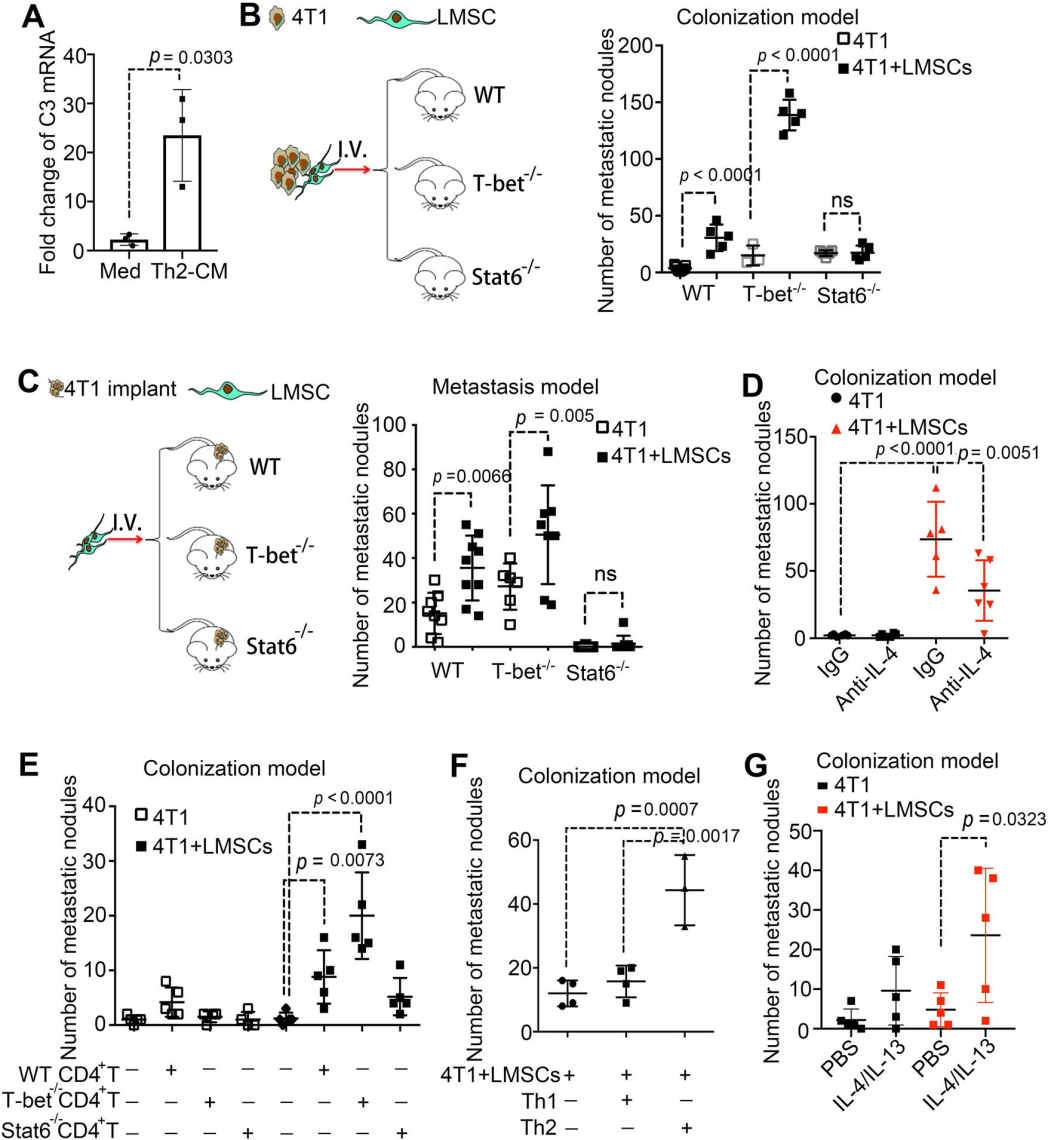
Th2-polarized microenvironment is required for sustaining the pro-metastatic effect of LMSCs. **A** Th2 cytokines increased the expression of C3. CD4^+^ T cells were induced into Th2 cells, which were then cultured in fresh medium for 12 h, to generate Th2-CM. LMSCs were treated with Th2 supernatant for 24 h and were examined for C3 expression. Med, culture medium. *n* = 3 individual experiments. **B** LMSCs failed to promote lung colonization in *Stat6*-deficient mice. LMSCs were co-injected intravenously with 4T1 cells and LMNs were counted on day 14. *n* = 7, 5, 3, 5, 5, and 5 mice. **C** LMSCs failed to promote metastasis in *Stat6*-deficient mice. 4T1 cells (4 × 10^5^) were implanted into the mammary gland fat pad and LMSCs (1 × 10^5^) were injected 10 days later. LMNs were counted on day 30. *n* = 8, 9, 6, 8, 9, and 9 mice. **D** Depletion of IL-4 attenuated the metastasis-promoting activity of LMSCs. After co-administration of LMSCs and 4T1 cells into BALB/c mice, anti-IL-4 (α-IL-4, 50 μg per mouse) or IgG was intraperitoneally every 2 days. LMNs were counted on day 14. *n* = 6, 6, 5, and 6 mice. **E** Adoptive transfer of CD4^+^ T cells from WT or T-bet-deficient mice conferred a metastasis-promoting activity of LMSCs. 4T1 cells were co-injected with or without LMSCs cells into nude mice. CD4^+^ T cells (4 × 10^6^) isolated from the indicated mice were injected into nude mice on day 2. LMNs were counted on day 14. *n* = 5, 5, 4, 4, 5, 5, 5, and 5 mice. **F** Adoptive transfer of Th2 cells conferred a metastasis-promoting activity of LMSCs. 4T1 cells were co-injected with LMSCs cells into nude mice. CD4^+^ T cells were induced into Th1 or Th2 cells, respectively, and injected (4 × 10^6^) on day 2. LMNs were counted on day 14. *n* = 4, 4, and 3 mice. **G** IL-4/IL-13 conferred LMSCs a pro-metastatic activity. Nude mice were co-injected with 4T1 and LMSCs (control or IL-4/IL-13-treated), and were treated with IL-4/IL-13 or PBS every 4 days. LMNs were counted on day 14. *n* = 5 mice each. All the data are presented as mean values ± SD. ns, not significant; *p* < 0.05, significant, using a one-way ANOVA with Sidak’s post test for **B**–**G**; using an unpaired, two-tailed Student’s *t*-test for **A**. Source data are provided as a Source Data file.

Considering that IL-13 and IL-4 are primarily derived from lymphocytes, especially Th2 cells, we monitored the Th2 cell population in the lungs of MMTV-PyMT mice and detected increased infiltration of CD4^+^ T cells and Th2 cells along with tumor progression (Supplementary Fig. 6A). Furthermore, when LMSCs were treated with Th2 cell culture supernatant, the C3 expression was significantly upregulated (Fig. 6A). Importantly, LMSCs treated with Th2 cell-conditioned medium displayed a greater metastasis-promoting ability than untreated LMSCs when co-injected with 4T1 into BALB/c mice (Supplementary Fig. 6B). To further confirm the effect of T cells on metastasis, we administered 4T1 cells and LMSCs into WT mice and T-cell-deficient nude mice, and observed that LMSCs failed to promote metastasis in nude mice (Supplementary Fig. 6C). These results demonstrate the importance of Th2 cells in promoting lung metastasis and are consistent with the reported lack of lung metastasis in MMTV-PyMT* Rag1*^−/−^ mice or *CD4*^−/−^*CD8*^−/−^ mice^20^. We next tested the function of LMSCs in *Stat6*^−/−^ mice and *T-bet*^−/−^ mice, respectively. *Stat6*^−/−^ mice are defective in the generation of Th2 cells^44^. In contrast, *T-bet*^−/−^ mice do not produce the Th1-type cytokine, but have elevated levels of IL-4 and IL-13 in their bronchial alveolar lavage fluids^45,46^. When i.v. co-injected with 4T1 cells, LMSCs showed no colonization-promoting function in *Stat6*^−/−^ mice. In a sharp contrast, there was a dramatic increase in lung colonization of 4T1 cells in *T-bet*^−/−^ mice as compared to WT mice (Fig. 6B). We next tested whether lung metastasis is influenced by the Th1- or Th2-skewed genetic background by implanting 4T1 cells into the mammary gland fat pads of WT, *Stat6*^−/−^, and *T-bet*^−/−^ mice, respectively (Fig. 6C). Half of the mice in each group also received LMSCs via i.v. injection 10 days after 4T1 implantation (Fig. 6C). Importantly, the metastasis-promoting effect of LMSCs was only observed in WT and *T-bet*^−/−^ mice, but not in the *Stat6*^−/−^ mice (Fig. 6C). Similarly, IL-4-specific antibodies administered intraperitoneally also blocked LMSC-aided colonization (Fig. 6D). To confirm that Th2 cells are required for sustaining the metastasis-promoting effect of LMSCs, we isolated CD4^+^ T cells from WT, *T-bet*^−/−^, and *Stat6*^−/−^ mice, respectively, and injected them, together with 4T1 cells and LMSCs, into nude mice. We observed that only CD4^+^ T cells from WT or *T-bet*^−/−^ mice significantly increased LMSC-aided metastasis, although CD4^+^ T cells from *T-bet*^−/−^ mice were more effective (Fig. 6E). Furthermore, we isolated CD4^+^ T cells and induced them to differentiate into Th1 and Th2 cells, respectively. Only Th2 cells, when co-injected with 4T1 cells and LMSCs, increased the number of metastatic nodules markedly (Fig. 6F). Similarly, we stimulated LMSCs with or without IL-4 and IL-13, and co-injected them together with 4T1 into nude mice. IL-4/IL-13-treated LMSCs increased the number of metastatic nodules in the lungs (Fig. 6G). Moreover, although administration of LMSCs resulted in an increased neutrophil accumulation when co-injected with 4T1 cells into WT or *T-bet*^−/−^ mice, it had no such effect in nude or *Stat6*^−/−^ mice (Supplementary Fig. 6D). The increase in neutrophil accumulation was more pronounced in *T-bet*^−/−^ mice than in WT mice (Supplementary Fig. 6D). Meanwhile, in *Stat6*^−/−^ mice, injection of LMSCs showed no NET formation-promoting function. In sharp contrast, more NETs were formed in *T-bet*^−/−^ mice than in WT mice (Supplementary Fig. 6E). These results support that Th2 cells, the source of IL-4/IL-13, are essential for sustaining neutrophil recruitment to the lungs and LMSC-aided tumor metastasis.

### C3 expression levels are higher in breast cancer patients

**Fig. 7:**
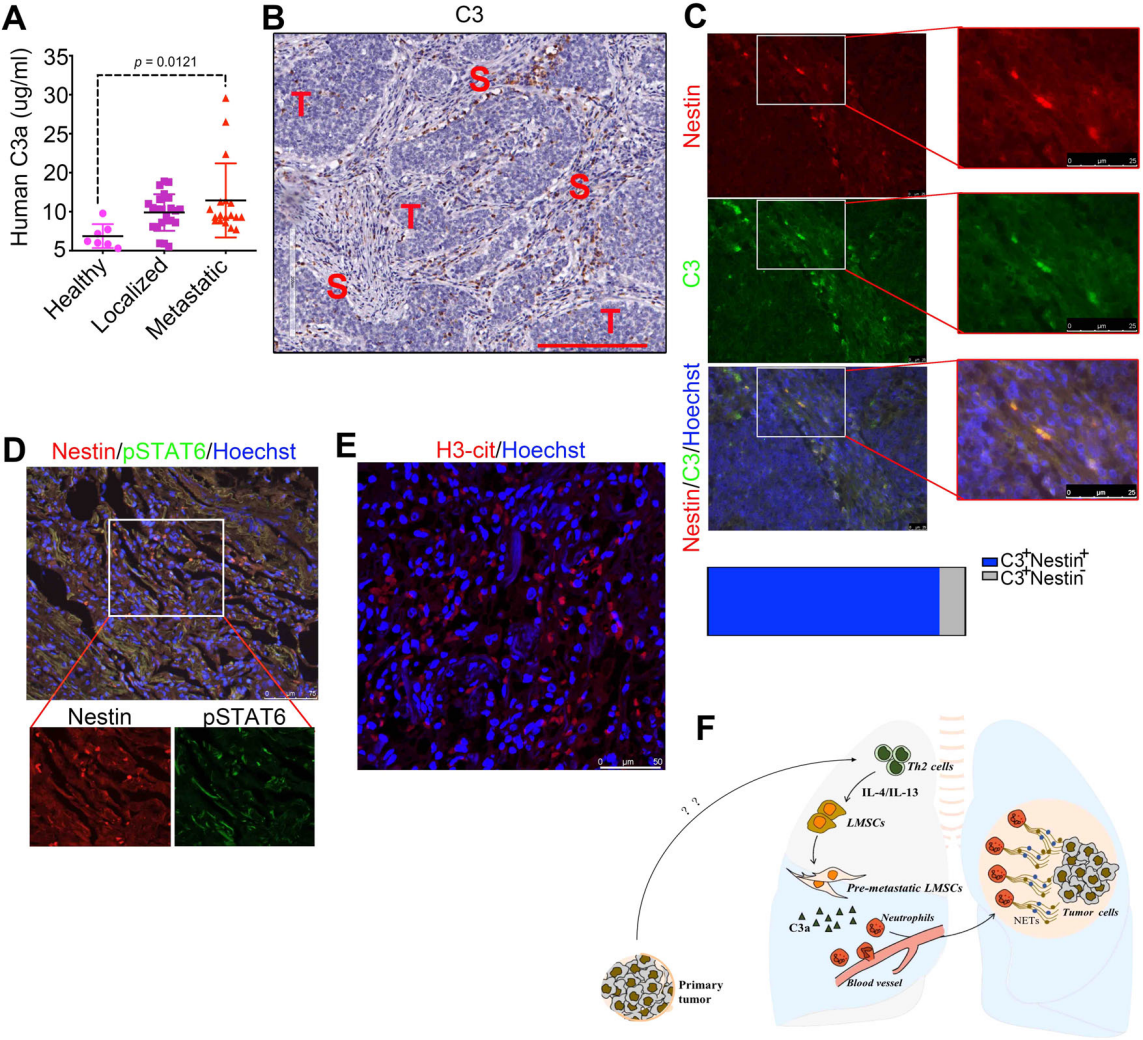
High C3 expression levels are associated with metastasis in breast cancer patients. **A** ELISA quantification of C3 in serum samples obtained from breast cancer patients with solid primary tumors or metastatic tumors. Final clinical diagnosis is indicated. *n* = 18 patients with metastasis, 23 patients with no metastasis, 7 healthy individuals. Data are presented as mean values ± SD, using a one-way ANOVA with Sidak’s post test. Source data are provided as a Source Data file. **B** Representative immunohistochemistry image showing the strong C3 (brown) staining in the stroma of breast cancer metastasized to the lung. Scale bar represents 200 μm. The images were representative of nine breast cancer patients with lung metastasis. **C** Representative immunofluorescence images showing the colocalization of Nestin (red) and C3 (green) in pulmonary tissues, in a patient with lung metastasis. Scale bar represents 25 μm. The percentages of C3^+^Nestin^+^ cells and C3^+^Nestin^−^ cells were shown in the horizontal bar. The images were representative of nine breast cancer patients with lung metastasis. **D** pSTAT6 (red) and Nestin (green) staining of pulmonary tissues from metastatic breast cancer site (MBC). Pulmonary tissue sections were examined for pSTAT6 and Nestin by IF. Scale bar represents 75 μm. The images were representative of nine breast cancer patients with lung metastasis. **E** H3-cit (red) staining of pulmonary tissues from metastatic breast cancer site (MBC). Pulmonary tissue sections were analyzed for H3-cit by immunofluorescence. Scale bar represents 50 μm. The images were representative of nine breast cancer patients with lung metastasis. **F** A schematic model depicting the mechanism by which LMSCs promote metastasis. The Th2 cytokines IL-4 and IL-13 induce LMSCs to express C3, which recruit neutrophils to the lung and induce NET formation. NETs retain the tumor cells and allow the newly arrived tumor cells to colonize.

To explore possible clinical relevance of our findings, we determined C3a levels in the sera of 41 breast cancer patients (23 localized and 18 with detectable metastasis) and 7 healthy controls by ELISA, and found that the C3a levels were statistically higher in metastasis group of patients (Fig. 7A). In addition, Kaplan–Meier Plotter analysis (http://www.kmplot.com) showed that high-C1QB (Complement C1q subcomponent subunit B) and low-C1INH (C1-inh, C1 esterase inhibitor) are correlated with poor distant metastasis-free survival in the breast cancer samples (Supplementary Fig. 7A). High-C1QB and low-C1INH possibly promote the activation of complement pathway and produce more C3a^47^. Furthermore, we examined the levels of C3 in lung metastatic specimens obtained from patients with metastatic breast cancers (MBC). C3 expression, as determined by immunohistochemistry, appeared to be more clustered in the stroma (Fig. 7B). In addition, most of the C3 signals were localized in LMSCs, which were marked by Nestin (Fig. 7C), suggesting that C3 may also be produced by LMSCs in cancer patients. Moreover, we examined the levels of pSTAT6 and found that pSTAT6 signals could be abundantly detected and were exclusively restricted to Nestin-positive cells, as determined by immunofluorescence, in MBC (Fig. 7D). Finally, we also detected H3-cit signals in MBC (Fig. 7E). These data suggest that C3 may be involved in lung metastasis of human breast cancer.

## Discussion

Metastasis is a key determinant of prognosis of cancer patients. It has been a long-standing question why a tumor cell can find its destination at a specific tissue site to form a secondary tumor. How the so-called PM tissue microenvironment or niche is established has recently attracted much interest. We here demonstrated the critical roles of LMSCs, Th2-type cytokines, and neutrophils in the formation of pulmonary PM niche and elucidated the mechanisms underlying the interaction between local MSCs and immune cells. It appears that prior to metastasis, local MSCs have already been reprogrammed by Th2 cytokines such as IL-4/IL-13, as characterized by the upregulation of complement C3. C3 is able to recruit more neutrophils to the PM sites and promote the formation of NETs through the C3–C3aR axis. With increased buildup of NETs, more circulating tumor cells may get trapped in the lung and are allowed to colonize (Fig. 7F). Our study established a mechanistic link between the local stromal cells—T cells, LMSCs, and neutrophils—in driving tumor metastasis.

Previously discovered role of MSCs in PM niche is limited to ECM remodeling. Some MSCs, acting as pericytes, regulate the extravasation of melanoma cancer cells to the bone marrow and liver via SDF1/CXCL12-CXCR4 signaling at their perivascular niche^48^. Irradiated MSCs could greatly enhance lung metastasis when infused i.v., via recruiting more macrophages^49^. This study revealed that LMSCs could remodel the PM niche by recruiting neutrophils and promoting NET formation via the C3–C3aR axis.

Numerous studies have indicated that neutrophils accumulate in the tumor microenvironment or metastatic niche to promote tumor metastasis^26,50–53^. How neutrophils are mobilized and recruited to PM niche was addressed in several studies. For example, tumor cell-derived G-CSF was shown to play an important role in the expansion, mobilization, and recruitment of neutrophils to the PM niche^54^. Lung epithelial cells, when exposed to tumor-derived exosomal RNAs, are also able to recruit neutrophils by secreting CXCL1, CXCL2, CXCL5, and CXCL12^14^. Consistent with previous reports, the level of G-CSF in serum is significantly increased at the PM stage (Fig. 5A), which may explain why neutrophils became more abundant along with tumor progression. Moreover, we demonstrated that C3 produced by LMSCs functions to promote neutrophil infiltration to the lungs for the formation of PM niche. In a mouse model of breast cancer metastasis, neutrophils in PM niche were shown to secrete leukotrienes that enrich metastasis-initiating cells^15^. NETs-associated proteases sequentially cleave the extracellular matrix protein laminin, allowing for proteolytic remodeling of laminin and leading to integrin-mediated signaling in cancer cells, finally activating proliferation of dormant cancer cells^22^. A recent study showed that the chromatin DNA component in NETs (NET-DNA) acts as a chemotactic factor to attract cancer cells by binding to CCDC24 during liver metastasis in breast and colon cancer patients^55^. These previous studies and the results we presented here, all demonstrate an important role for neutrophils and NETs in metastasis. The identification of C3 as a critical regulator of NETs is consistent with the reports that *C3aR*^−/−^ neutrophils could not form the NETs when treated with lipopolysaccharides^41^ and NET formation requires both TLR2 and C3^36^. This function of C3 in the regulation of neutrophils may have a broad implication in the biology of inflammation.

It was reported that a Th2 immune skewing in tumor stroma promotes tumor progression and correlates with reduced survival in pancreatic cancer^56^. Melanoma was also reported to be associated with Th2-mediated chronic inflammation^57^. In the MMTV-PyMT mice, the levels of type 2 inflammation cytokines such as IL-4 (PE) and IL-13 (serum) increased with tumor progression. Importantly, there were more lung metastases of 4T1 breast cancer cells in the Th1-deficient (Th2-skewed) genetic background. IL-4-expressing CD4^+^ T cells were previously shown to promote pulmonary metastasis of breast cancer by directly acting on tumor-associated macrophages, which enhance metastasis through activating epidermal growth factor receptor signaling in cancer cells^20^. We here showed that IL-4 and IL-13 could directly act on MSCs and increase the expression of C3 through STAT6 signaling, and C3a subsequently functions to recruit neutrophils to the lungs and to promote the formation of NETs and metastasis. It was reported that allergen-induced pulmonary inflammation, a Th2-skewed condition, could result in more than threefold increase in lung metastasis of i.v.-injected B16-F10 melanoma cells^58^. Interestingly, the increased metastasis associated with allergenic respiratory inflammation was prevented by corticosteroids, a general immunosuppressive drug. Furthermore, the incidence of asthma was higher among patients with breast cancer metastasis^58^. Together, these findings suggest that Th2 polarization in lung may represent a significant risk factor for lung metastasis. Although these observations clearly indicate a link between Th2 polarization and lung metastasis, how the primary tumors induce Th2 skewing in the lung remains to be elucidated. It has been shown that that the CCL5/CCR3 signaling pathway promotes lung metastasis of breast cancer in PyMT mice by inducing Th2 polarization of CD4^+^ T cell^59^, although the cellular source of CCL5 is unknown. Future studies should reveal whether primary breast tumors directly produce CCL5 to elicit the Th2 immune response or promote the generation of CCL5 by other tissues or cells.

It should be noted that the main conclusion of this study is based on the observation that LMSCs at different stages of tumor development in MMTV-pyMT (FVB strain) are functionally distinct in their pro-metastatic ability when re-introduced into MMTV-pyMT mice. These MSCs could also facilitate the metastasis of transplanted 4T1 breast cancer cells in BALB/c mice. Introducing allogenic MSCs may seemingly confound the elucidation of the tumor-promoting effect of MSCs, as they may trigger immune rejection responses by the hosts and reduce tumor growth and metastasis. However, it is well known that MSCs only express low levels of MHC I and no MHC II, thus having minimal immunogenic effect, if any. Quite on the contrary, in mice and humans, MSCs, irrespective of the MHC haplotypes of the donors, have been successfully employed to treat graft-versus-host-disease^60,61^. Allogeneic human bone marrow and umbilical cord-derived MSCs have been widely used for the treatment of many autoimmune diseases world wide^23^. Due to their immunosuppressive nature, MSCs derived from different genetic backgrounds, even those from different species, have not been found to elicit immediate rejection response. In fact, had MSCs induced rejection response, the tumor growth and metastasis would have been reduced after MSC infusion. Instead, we observed increased lung metastasis of 4T1 tumor cells upon MSC infusion. Moreover, the pro-metastatic effect of MSCs was demonstrated to require their increased production of C3 that is endowed by Th2 cytokines. Thus, the infused MSCs can presumably persist in the host long enough to exhibit the Th2-dependent pro-metastatic response. It should also be noted that more metastasis of the implanted 4T1 cells occurred in the Th2-skewed *T-bet*^*−/−*^ than in WT mice even in the absence of exogenous MSCs, whereas the opposite was true in the Th1-skewed *Stat6*^*−/−*^ mice (Fig. 6C). This result provides further support for a pro-metastatic role of the Th2 cytokine–Stat6 axis.

In conclusion, our present study provides insights into the role of local MSCs in the establishment of the PM niche. We demonstrated that under the influence of Th2 cytokines, such as IL-4/IL-13, LMSCs are activated to express high levels of C3, which promotes neutrophil recruitment, NET formation, and the subsequent lung metastasis. Therefore, targeting the Th2 cytokine–STAT6–C3–NETs axis may represent a potential strategy for the prevention and management of lung metastasis. Although further investigation is needed to fully understand the implications of our findings, the IL-4/IL-13-STAT6-C3 axis is likely to operate in other pathological conditions, especially when LMSCs or other stromal cells are involved.

## Methods

### Mice

BALB/c (BALB/c J), MMTV-PyMT (FVB/N-Tg (MMTV-PyVT) 634Mul/J), FVB (FVB/NJ), Nude (NU/J), *Stat6*^*−/−*^ (C.129S2-*Stat6*^*tm1Gru*^/J), *T-bet*^*−/−*^ (C.129S6-*Tbx21*^*tm1Glm*^/J), *C3*^*−/−*^ (B6.129S4-*C3*^*tm1Crr*^/J), and *C3aR*^*−/−*^ (C.129S4-*C3ar1*^*tm1Cge*^/J) mice were obtained from the Jackson Laboratory and bred in a specific pathogen-free animal facility of Soochow University (temperature 22 °C, humidity 59 rH using a 12/12 h dark/light cycle). The animal protocols for the experiments described in this study were approved by the Ethical Committee of Soochow University.

### Human subjects

All tissues and sera were obtained with informed consent compliance with the Ethical Committee of the First Affiliated Hospital of Soochow University. All patients provided written informed consent prior to study entry. Clinical details were recorded in Supplementary Tables 1 and 2.

### Cell line

Murine 4T1 (ATCC, CRL-2539), a mammary gland tumor cell line of BALB/cfC3H origin, was kindly provided by Stem Cell Bank, Chinese Academy of Science. This cell line was cultured in Dulbecco’s modification of Eagle’s medium (DMEM) supplemented with 10% bovine serum albumin (fetal bovine serum (FBS)), 2 mM glutamine, 100 U/ml penicillin, and 100 g/ml streptomycin (all from Invitrogen)

### Lung metastasis assays

#### Staging of tumor development in MMTV-PyMT mice

Tumor progression in MMTV-PyMT mice was divided into three stages as follows: (i) A referred to 7- to 8-week-old mice with the presence of few primary tumors; (ii) PM referred to 10- to 11-week-old mice without tumor in the lung; and (iii) M to those at 13- to 14-week-old with tumor metastasized to the lung.

#### Metastasis of primary mammary tumors

LMSCs (1 × 10^5^) were administrated i.v. into MMTV-PyMT mice at the PM stage (7- to 8-week-old, female). On day 35 after LMSCs inoculation, tumor nodules on the lung were counted and examined.

#### Metastasis of 4T1 cells implanted in mammary fat pad

4T1 cells (4 × 10^5^) were injected into BALB/c mice (6–8 weeks old, female) in the mammary gland fat pad. After 10 days, LMSCs (1 × 10^5^) isolated from mice at different tumor stages were respectively injected i.v. to the 4T1 tumor-bearing mice. On day 30 after tumor cell inoculation, tumor nodules in the lung were counted and examined using Bouin’s solution (Sigma HT10132-1L).

#### Colonization of 4T1 cells injected i.v

4T1 cells (5 × 10^4^ [low dose] or 1 × 10^5^ [high dose]) were co-injected with LMSCs (1 × 10^4^) into BALB/c mice (6–8 weeks old, female) through i.v. injection. On day 14 after tumor cells inoculation, tumor nodules in the lung were counted and examined. Each experimental group included at least three mice. All metastasis experiments were replicated at least twice.

### Isolation of LMSCs from the lung tissue

LMSCs were established from the lungs of MMTV-PyMT mice at different tumor stages: A stage (7- to 8-week-old mice, presence of small primary tumor mass), PM stage (10- to 11-week-old mice, presence of large primary tumor mass, but with absence of metastasis), and M stage (13- to 14-week-old mice, presence of metastasis in the lung). Briefly, the lung tissue was digested with type II collagenase (0.5 mg/ml) and hyaluronidase (0.1 mg/ml) at 37 °C for 2 h, the single cells were then cultured in DMEM medium supplemented with 10% FBS, 2 mM glutamine, 100 U/ml penicillin, and 100 g/ml streptomycin (all from Invitrogen). Non-adherent cells were removed after 24 h and adherent CD45^−^ cells were purified by immunomagnetic separation using mouse CD45 microbeads (Miltenyi Biotec, 130-052-301), and then maintained in culture medium (three dishes/mouse), which was replaced every 3 days. All LMSCs were confirmed to have no hematopoietic stem (progenitor) cells and lineage cell surface makers. MSCs became more homogeneous after two passages and beared the following markers: CD29^+^ CD44^+^ CD140a^low^Nestin^+^ Sca1^low^Lineage^−^CD11b^−^CD31^−^ CD34^−^ CD45^−^. The “stemness” of LMSCs was determined by their capability to undergo osteogenic and adipogenic differentiations under appropriate differentiation-inducing conditions.

### Flow cytometry

Characterization of the surface markers in the various cell types in different tissues were performed by flow cytometry. Lung tissue was digested using type II collagenase (Thermo Fisher 17101015) in DMEM medium for 1 h at 37 °C and the digested samples were then filtered through a 70 μm cell strainer, washed, and resuspended in phosphate-buffered saline (PBS) supplemented with 2% bovine serum albumin (FBS). Total cell counts were determined using flow cytometry. For surface marker analysis, cells isolated from lung tissues were suspended in staining buffer (PBS, 5% FBS) at a concentration of 1 × 10^6^ cells/ml and 50 μL of suspension was incubated with fluorescently labeled antibodies (all 1 : 100) in a 96-well plate for 30 min at 4 °C. After washing, the cells were resuspended for analysis or further stained for intracellular analysis. The antibodies used for flow cytometry were as follows: fluorescein isothiocyanate (FITC) anti-CD44 (eBioscience, 11-0441-85), 7-Aminoactinomycin D (eBioscience 00-6993-50), PE anti-mouse CD45 (BioLegend 147712), pacific Blue anti-mouse CD3 (BioLegend 100214), BV510 anti-mouse CD19 (BD Bioscience 562956), BV605 anti-mouse CD19 (BioLegend 115540), APC-H7 anti-mouse CD4 (BD Bioscience 560181), FITC anti-mouse CD4 (eBioscience 11-0041-85), APC anti-mouse CD8a (BioLegend 100712), FITC anti-mouse CD11b (eBioscience 11-0112-85), APC anti-mouse F4/80 (eBioscience 17-4801-82), PE anti-mouse Ly6G/Ly6C (BD Bioscience 12-5931-83), PE-Cy7 anti-mouse Sca1 (BioLegend 108114), PE anti-mouse IL-4 (eBioscience 12-7041-82), and FITC anti-mouse-inteferon-γ (IFNγ) (eBioscience 11-7311-81). The following antibodies were used for LMSC identification: PE anti-mouse CD29 (eBioscience 12-0291-81), PE anti-mouse CD44 (eBioscience 12-0441-83), PE anti-mouse CD140a (eBioscience 12-1401-81), eFluor 450 anti-mouse Lineage (eBioscience 88-7772-72), and Rat anti-mouse Nestin (Abcam ab81462). Major reagents used for intracellular assay are as follows: inomycin (Sigma I3909-1ML), PMA (Sigma P1585-1MG), and Protein Transport Inhibitor (BD Bioscience 554724). Fluorescence intensity was measured by flow cytometry (Cytoflex, Beckman Coulter). Flow Jo v10 and Cytexpert software were used for data analysis.

### RNA-seq of LMSCs and bioinformatics analysis

LMSCs isolated from MMTV-PyMT mice at different tumor stages (*n* = 3 for each stage) were subjected to RNA-seq analysis. The accession number for RNA-seq data deposited in NCBI Gene Expression Omnibus is GEO: GSE125591 (*n* = 2) and GSE179315 (*n* = 1). We used limma (3.46.0) to deal with the difference of Reads between two experiments and analyzed the data by pheatmap (1.0.12).

### Quantitative real-time PCR

Total RNA was isolated from cell pellets using Trizol reagent (Thermo Fisher Scientific, 15596-026). cDNA synthesis was carried out using PrimeScript™ RT Master Mix (TaKaRa Biotech, RR036A) with random hexamer primers. Levels of mRNA for genes of interest were quantified by real-time PCR (ABI Quant Studio 6, Life) using SYBR Green Master Mix (Thermo Fisher Scientific, 4472920). Thermocycling included an initial incubation at 95 °C for 20 s, followed by a two-step PCR program of 95 °C for 15 s and 60 °C for 60 s, for a total of 45 cycles. The total amount of mRNA was normalized to endogenous β-actin mRNA using the ΔCt method. Oligonucleotides are listed in Supplementary Table 3.

### Western blotting

Cells or lung tissue samples were lysed with RIPA buffer containing protease inhibitors and protein concentrations determined by BCA Protein Assay Kit (Thermo Fisher Scientific, 23227). Denatured proteins were separated by SDS-polyacrylamide gel electrophoresis and transferred to polyvinylidene difluoride membrane (Merck Millipore, IPVH00010). Blottings were incubated with primary Abs overnight at 4 °C and then with secondary Abs for 1 h at room temperature. The following primary antibodies were used: Mouse monoclonal anti-β-actin antibody (Sigma, A5441) (1 : 1000), Rabbit anti-C3 (Abcam ab200999) (1 : 500), Rabbit polyclonal to Histone H3 antibody (citrulline R2 + R8 + R17)-ChIP Grade (Abcam, ab5103) (1 : 500), and Rabbit anti-pSTAT6 (Cell Signaling Technology 56554) (1 : 500).

### Detection of cytokines

The levels of cytokines and chemokines in culture supernatants, PE, or serum were assayed by multiplexed bead array immunoassay using Luminex Technology according to the manufacturer’s protocol (Bio-Plex, M60009RDPD). Other ELISA kits: mouse anti-IL4 ELISA kit and human anti-C3a ELISA kit were from Thermo Fisher Scientific; mouse anti-C3 ELISA kit was from Abcam.

### Immunofluorescence

Lung tissues were washed in PBS and fixed in 10% PBS-buffered formalin for 24 h. Tissues were then embedded in optimal cutting temperature compound, and 5–6 μm sections were cut by Microtome Cryostat (Leica CM3050S, Leica Biosystems), and stained with antibody according to the protocol provided by the manufacturer. Cells, cultured on the microscope cover glass, were washed in PBS and fixed in 10% PBS-buffered formalin for 15 min, and then stained with antibodies according to the protocol provided by the manufacturer. The sections were examined using a laser scanning confocal microscopy (Leica Biosystems). Primary antibodies (1 : 200) were applied as follows: Rat anti-mouse C3 (Abcam ab11862), Rabbit anti-C3 (Abcam ab200999), Rat anti-mouse Nestin (Abcam ab81462), Rat anti-mouse Ly6G (Abcam ab25377), Rabbit anti-human C3 (Abcam ab97462), Mouse monoclonal anti-human Nestin (Abcam ab22035), Rabbit anti-Histone H3-cit (Abcam ab5103), and Rabbit anti-Myeloperoxidase (Abcam ab208670). The following secondary antibodies (1 : 1000) were used: Alexa Fluor 488 Goat anti-Rat IgG (Abcam ab150157), Alexa Fluor 488 Goat anti-Rabbit IgG (Abcam ab150077), Alexa Fluor 555 Donkey anti-Rabbit IgG (Beyotime A0453), Alexa Fluor 647 Goat anti-mouse IgG (Beyotime A0473), and Alexa Fluor 594 Goat anti-Rat IgG (Abcam ab150160).

### Immunohistochemistry

Lung tissues were washed in PBS and fixed in 10% PBS-buffered formalin for 24 h. Tissues were then embedded in paraffin and 5–6 μm sections were cut, deparaffinized, and stained with antibody according to the protocol provided by the manufacturer. Rabbit anti-human C3 (Abcam ab97462) (1 : 500) was used as a primary antibody.

### RNA interference

Stable and transient knockdown of gene expression was achieved by using targeted shRNA or small interfering RNA sequences, respectively. A nonspecific SCR sequence was used as a control in both cases. LV3-shNC or LV3-shC3 virus (GenePharma, China) was used to infect expanded PM-LMSCs (at passage 3) in vitro and positive cells were selected using puromycin starting from 48 h post infection. Puromycin-resistant PM-LMSCs, which were FITC-positive, were analyzed for knockdown efficiency.

### Mouse recombinant C3a

mC3a (25 μg per mouse) (R&D Systems, 8085-C3-025) was administered i.v. into 4T1 tumor-bearing mice (BALB/c, female, 6–8 weeks old) on days 1 and 7, and then lung metastatic nodules were counted on day 14.

### Depletion of neutrophils

After co-administration of PM-LMSCs (1 × 10^4^) with 4T1 cells (5 × 10^4^) into BALB/c mice (6–8 weeks old, female), anti-Ly6G (α-ly6G, 50 μg per mouse) (BioLegend, 127649) or IgG (BioLegend, 400544) was administered intraperitoneally on days 2, 4, 6, 8, 10, and 12. Lung metastatic nodules were counted on day 14.

### DNase I treatment

After LMSCs and 4T1 cells were co-injected into BALB/c mice (6–8 weeks old, female), DNase I (400 U per mouse) was administered i.v. on days 1, 4, 7, and 10, and lung metastatic nodules were counted on day 14.

### Neutrophil isolation

Single-cell suspensions of bone marrow were obtained and neutrophils were isolated using magnetic beads sorting (EasySep™ Mouse Neutrophil Enrichment Kit, STEM CELL, Cat#19762).

### Transwell chemotaxis assay

Neutrophil recruitment was assessed using a transwell assay. Neutrophils (1 × 10^5^ cells) were placed on the upper compartment and were separated from the lower compartment by a 10 μm thick poly-carbon membrane with 3.0 μm pores. LMSCs (1 × 10^4^ cells) or mC3a (1 μg/ml) were placed on the lower compartment. After co-culturing for 6 h, neutrophils in the lower compartment were enumerated.

### In vitro T-cell differentiation

CD4^+^ T cells from spleens of 6- to 8-week-old mice were purified by negative magnetic selection (Stem Cell, 19852). Th1 conditions: cells (1 × 10^6^ cells/ml) were activated by plastic-bound anti-CD3 in the presence of soluble anti-CD28 and cultured with IL-2 (200 U/ml), IL-12 (10 ng/ml), and anti-IL-4 (10 μg/ml) for 48 h. Th2 conditions: cells (1 × 10^6^ cells/ml) were activated by plastic-bound anti-CD3 in the presence of soluble anti-CD28 and cultured with IL-2 (200 U/ml), IL-4 (5 ng/ml), and anti-IFNγ (10 μg/ml) for 48 h. The basic medium is RPMI-1640 medium supplemented with 10% heat-inactivated FBS, 2 mM glutamine, 100 mg/ml streptomycin, and 100 U/ml penicillin.

### Statistical analysis

All data are represented as mean values ± SD. The GraphPad Prism 8 software was used for the statistical analyses. Statistical significance was assessed by unpaired and two-tailed Student’s *t*-test when only two groups were compared or one-way analysis of variance with Sidak’s post test when more than two groups were compared. ns, not significant; *p* < 0.05, significant.

### Reporting summary

Further information on research design is available in the Nature Research Reporting Summary linked to this article.

## Supplementary information

Supplementary information

Reporting Summary

## Data Availability

LMSCs sequencing data have been deposited in the GEO under accession number GSE125591 and GSE179315. The remaining data are available within the Article and Supplementary Information. Source data are provided with this paper.

## Source data

Source data
