# Supplementary material for: Lung mesenchymal stromal cells influenced by Th2 cytokines mobilize neutrophils and facilitate metastasis by producing complement C3
Source: Nat Commun. 2021 Oct 27;12:6202. doi: 10.1038/s41467-021-26460-z (PMC8551331; doi:10.1038/s41467-021-26460-z)
Supplement: Supplementary file 1 — Supplementary information [file 41467_2021_26460_MOESM1_ESM.pdf]

Supplementary Information

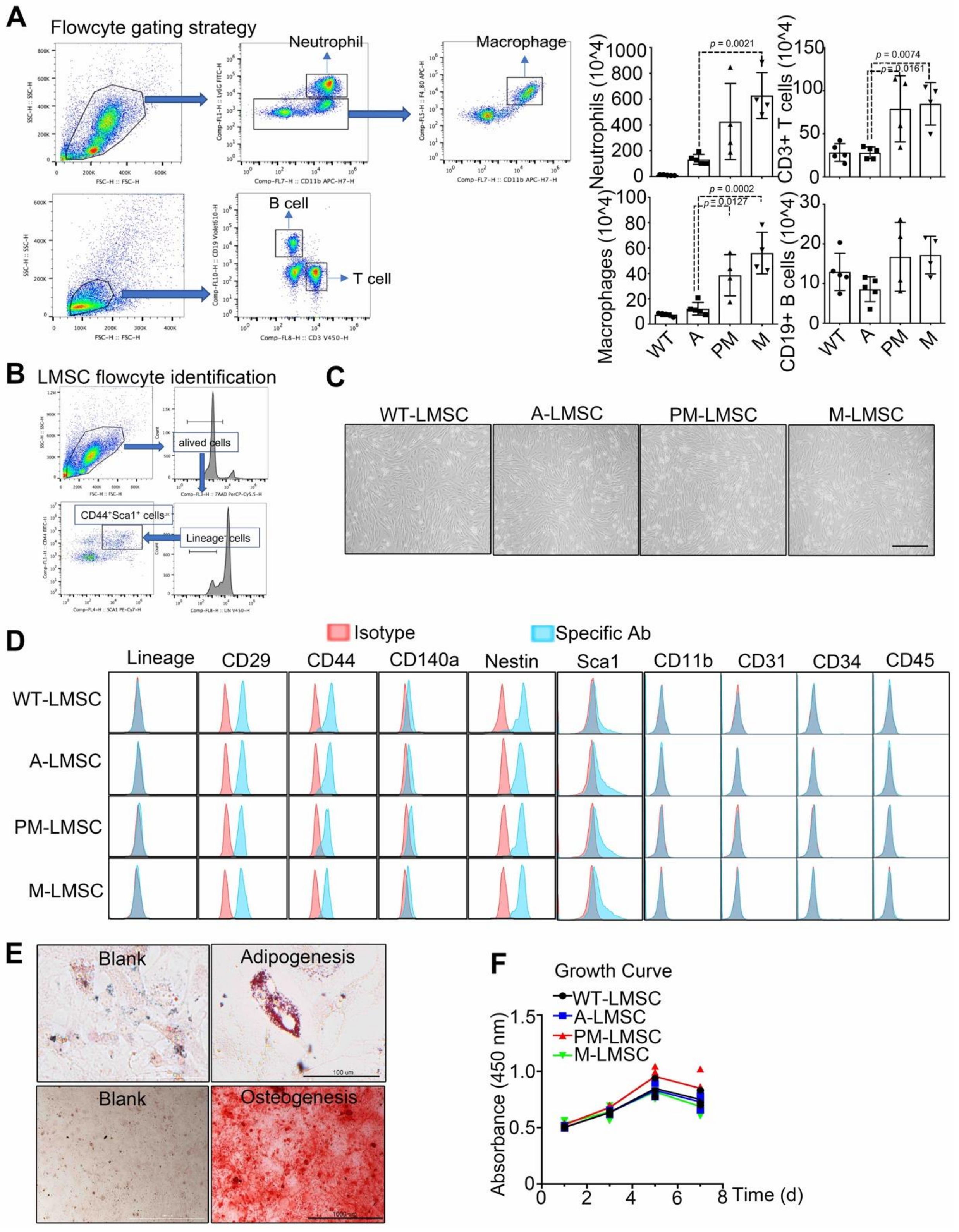

Supplementary Figure 1. Enumeration of immunocytes, and characterization of LMSCs during tumor progression, related to Fig. 1.

(A) FACS gating strategies. The lungs were isolated from different stages of MMTV-PyMT mice and the immunocyte types were analyzed and enumerated. Neutrophils were identified as $CD11b^{+}Ly6G^{+}$. Macrophages were identified as $CD11b^{+}F4/80^{+}Ly6G^{-}$. T cells were identified as $CD3^{+}$ and B cells were identified as $CD19^{+}$. *n* = 5, 5, 4, 4 mice. Data are presented as mean values +/- SD. $p < 0.05$, significant, using a one-way ANOVA with Sidak post-test. Source data are provided as a Source Data file.

(B) Identification of LMSCs by flow cytometry. LMSCs were identified by $7AAD^{-}Lineage^{-}CD44^{+}Sca1^{+}$.

(C) Representative morphology of WT-LMSCs, A-LMSCs, PM-LMSCs and M-LMSCs. The scale bar represents 500 μm. The images were representative of three independent experiments.

(D) WT-LMSCs, A-LMSCs, PM-LMSCs and M-LMSCs are phenotypically similar. After isolation from lungs, WT-LMSCs, A-LMSCs, PM-LMSCs and M-LMSCs, each from passage 3, were analyzed for the indicated markers by immunofluorescence staining and flow cytometry.

(E) Representative differentiation capability of LMSCs. LMSCs were differentiated into adipocytes and osteoblasts under defined conditions. The upper scale bar represents 100 μm. The bottom scale bar represents 1000 μm. The images were representative of three independent experiments.

(F) WT-LMSCs, A-LMSCs, PM-LMSCs and M-LMSCs possess similar proliferation capacity. WT-LMSCs, A-LMSCs, PM-LMSCs and M-LMSCs (1×10^{3}) were cultured in 96-well plates and detected on days 1, 3, 5, and 7 by CCK8 detecting. Source data are provided as a Source Data file.

A

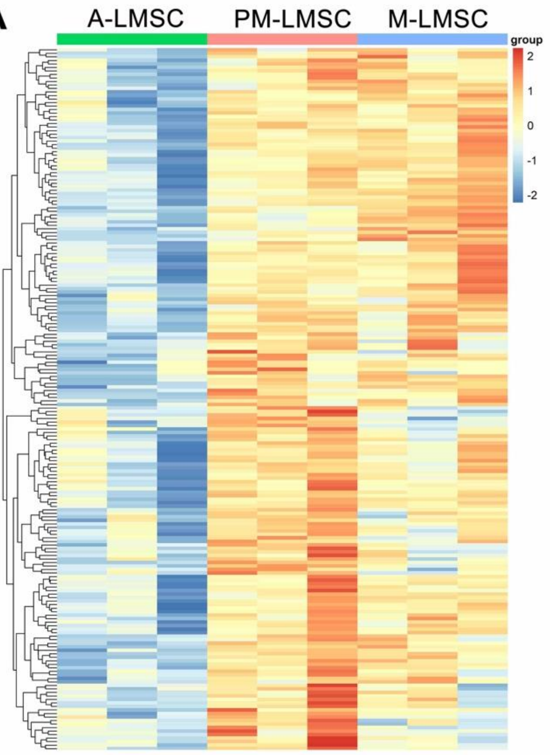

Upregulated genes

C3	Gpm6a	Gm12002	Edn2	Scube2
Ezr	2310030G06Rik	A630052C17Rik	Gm11960	Bhlha15
C1s1	Scube3	Col4a3	Gm42532	2810407A14Rik
Upk1b	Tnfsf18	Vtcn1	Inpp5j	Cdkl4
Gdf6	Mamdc2	Casq2	Scara5	Gm19721
Aldh1a1	Adgrg2	Fermt1	Bco1	Lppr5
Trim16	Hnrnpa3	RP23-200M5.7	RP23-132J21.5	Ermap
Atp1b1	Tmem35	Dnajc6	Amica1	BC021767
Bst1	Mapk4	Esyt3	5830418P13Rik	Serpina11
Tet3	Scx	Egf	Slitrk6	Gm11413
Gm38394	Gipc2	Cd59b	Tex26	Cd300e
Lgr6	Tfcp2l1	Lor	Gm43372	1700092M07Rik
Cxadr	Prnd	Rnasel	Hcn1	Abcc12
9930014A18Rik	Cdh1	Hmgcll1	Caskin1	Ndp
Mpzl2	Cfap45	Gm16759	Dock3	Cfap46
Cd34	Trpc6	Sprr2g	Gm16907	Gm6498
Olr1	Col4a4	Trpc4	Rab26os	Tie1
Serpinb2	Prg4	Gpbar1	Grip1	Gm16098
Mtss1	Hoxc13	Plcd4	Dmkn	Gm26945
Tnfaip8	Rsad2	Gprc5c	Clec4a2	9630013D21Rik
Il17rd	Pcdhb19	Gm10010	Gm10044	RP23-20L3.2
Gsta3	Fam184a	Nudt10	RP24-470M22.10	Gm28151
Smpd3	Rhpn2	Gm11127	Gm3052	Dgki
Thsd4	Slc1a3	Pde1c	Gm10814	Hist1h4k
Ncf2	Slfn10-ps	Filip1	Slc6a5	Nat1
Klhdc8a	Cttnbp2	Nefm	Gm38357	Gm15954
Mylk2	Vit	Klhl3	Gm10800	RP23-89P21.4
Plce1	Dync1i1	Lce1f	Myom3	Gm10605
Zbtb34	Sox6	A230020J21Rik	Astn1	Cbln1
Pcolce2	Tlr13	Crispld1	A930029G22Rik	Gm15704
Axin2	F2rl2	Pou2f2	Mccc1os	Gm9767
Dyx1c1Ccpg1	B230217C12Rik	Kank4	Gm43445	Gm13446
Fam180a	Gm28424	Lhx4	Clec4e	A730020E08Rik
Ugt1a6a	Tmem56	Gm43128	Kctd8	Platr16
St3gal6	Atp13a5	Gja3	Gm42936	Gm43101
Wnt2	Unc45b	Gm37565	Vmn2r79	Txndc2
Cd38	Ppp2r2b	Gm26760	Chrna5	Sp7
9330182L06Rik	Htr2b	Pglyrp3	Chst3	Stk32a
Tnfaip6	Lef1	B130024G19Rik	Adtrp	E130006D01Rik
Kprp	Cntn2	Deptor	Rhoh	Abcc2

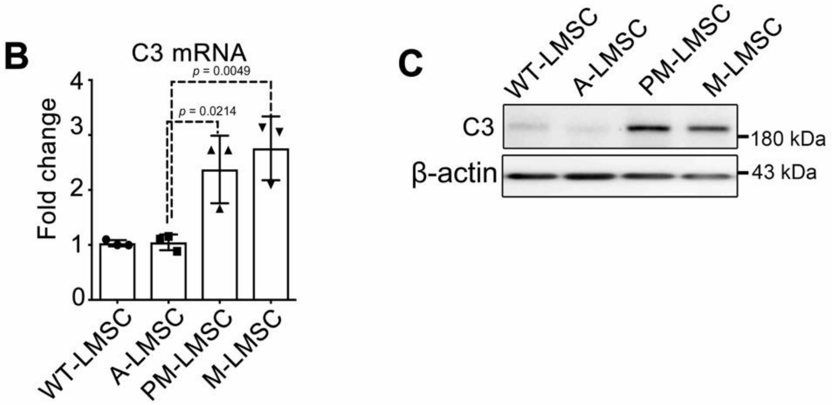

Supplementary Figure 2. C3 is highly expressed in PM-LMSCs and M-LMSCs, related to Fig. 2.

(A) Upregulated expression genes cluster of PM-LMSCs or M-LMSCs versus A-LMSCs. The list of upregulated genes is showed in the table. *n* = 3 mice for each stage.

(B) mRNA of LMSCs derived from the lungs of MMTV-PyMT mice at different tumor stages were detected for the expression of C3 by qRT-PCR. n=3 biologically independent cell samples. Data are presented as mean values +/- SD, $p < 0.05$, significant, using a one-way ANOVA with Sidak post-test. Source data are provided as a Source Data file.

(C) Expression of C3 in WT-LMSCs, A-LMSCs, PM-LMSCs and M-LMSCs. Proteins from $5x10^4$ WT, A-, PM-or M-LMSCs grown in 12-well plates were collected at 24 hr and detected for C3 expression by western blotting analysis. The images were representative of two independent experiments. Source data are provided as a Source Data file.

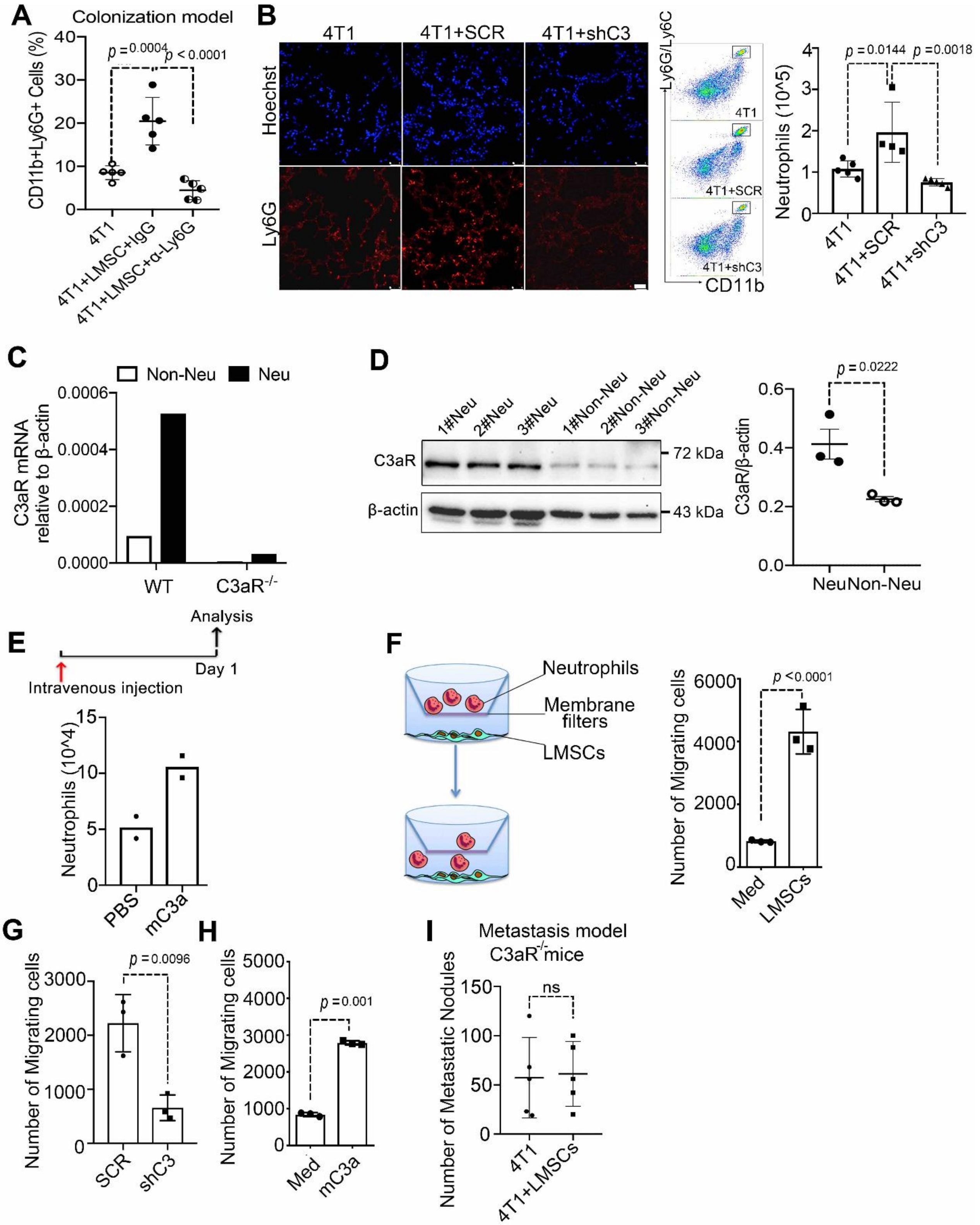

A
Colonization model
CD11b+Ly6G+ Cells (%)
p = 0.0004
p < 0.0001
4T1
4T1+LMSC+IgG
4T1+LMSC+α-Ly6G
B
4T1
4T1+SCR
4T1+shC3
Hoechst
Ly6G
Ly6G/Ly6C
CD11b
Neutrophils (10^5)
p = 0.0144
p = 0.0018
C
Non-Neu
Neu
C3aR mRNA relative to β-actin
WT
C3aR-/-
D
1#Neu
2#Neu
3#Neu
1#Non-Neu
2#Non-Neu
3#Non-Neu
C3aR
β-actin
72 kDa
43 kDa
C3aR/β-actin
p = 0.0222
Neu
Non-Neu
E
Analysis
Day 1
Intravenous injection
Neutrophils (10^4)
PBS
mC3a
F
Neutrophils
Membrane filters
LMSCs
Number of Migrating cells
p <0.0001
Med
LMSCs
G
Number of Migrating cells
p = 0.0096
SCR
shC3
H
Number of Migrating cells
p = 0.001
Med
mC3a
I
Metastasis model
C3aR-/- mice
Number of Metastatic Nodules
ns
4T1
4T1+LMSCs

Supplementary Figure 3. Knockdown of C3 in LMSCs reduces neutrophils infiltration, related to Fig. 3.

(A) After co-administration of LMSCs together with 4T1 cells into BALB/c mice, anti-Ly6G (α-ly6G, 50 μg per mouse) or control IgG was administered intraperitoneally on days 2, 4, 6, 8, 10, and 12. Lung tissues were stained with anti-Ly6G for the identification of neutrophils, which were also analyzed by flow cytometry ($CD11b^{+}Ly6G^{+}$) on day 14. n = 5 mice each.

(B) 4T1 cells were co-injected with SCR or shC3 PM-LMSCs at a 5:1 ratio (5×10^4 4T1 cells and 1×10^4 LMSCs) into BALB/c mice. Lung tissues were stained with anti-Ly6G for the identification of neutrophils, which were also analyzed by flow cytometry on day 14. *n* = 5, 4, 5 mice. The scale bar represents 25 μm.

(C) Neutrophils from WT and $C3aR^{-/-}$ mice were isolated from bone marrow, and the rest of the nucleated bone marrow cells were considered as Non-Neu cells. All cells were detected for C3aR at the mRNA level.

(D) Neutrophils from WT mice were isolated from bone marrow, and the rest of the nucleated bone marrow cells were considered as Non-Neu cells. All cells were detected for C3aR at the protein level. n = 3 mice each.

(E) Recombinant murine C3a (mC3a) recruites neutrophils to the lungs. mC3a (1.25 mg/kg) or PBS was injected into BALB/c mice respectively. The mice were euthanized for the examination of neutrophils in the lungs by flow cytometry. n = 2 mice each.

(F) LMSCs recruit neutrophils in vitro. Neutrophil recruitment was assessed using a transwell assay. Neutrophils were placed on the upper compartment and LMSCs on lower compartment. After co-culturing for 6 h, neutrophils in the lower compartment were enumerated by flow cytometry. n=3 independent experiments.

(G) (H) C3 mediates LMSCs-induced neutrophil recruitment. Neutrophils were

seeded on the upper compartment and supernatant from LMSCs (D)or mC3a (E) in lower compartment. After co-culturing for 6 h, neutrophils in the lower compartment were enumerated by flow cytometry. n=3 independent experiments.

(I) 4T1 cells (4 x 10^5) were implanted into the mammary gland fat pads of *C3aR*$^{-/-}$ mice. After 10 days, LMSCs (1 x 10^5) were administered into these tumor-bearing mice by intravenous injection. The lung metastatic nodules were counted on day 30. *n* = 5 mice each.

For Supplementary Fig. 3A-B, 3D and 3F-I, the data are presented as mean values +/- SD. ns, not significant; $p < 0.05$, significant, using a one-way ANOVA with Sidak post-test for Supplementary Fig.3A-B; using an unpaired, two-tailed, Student's t-test for Supplementary Fig.3D, 3F-I. Source data are provided as a Source Data file for Supplementary Fig.3A-B, D-I.

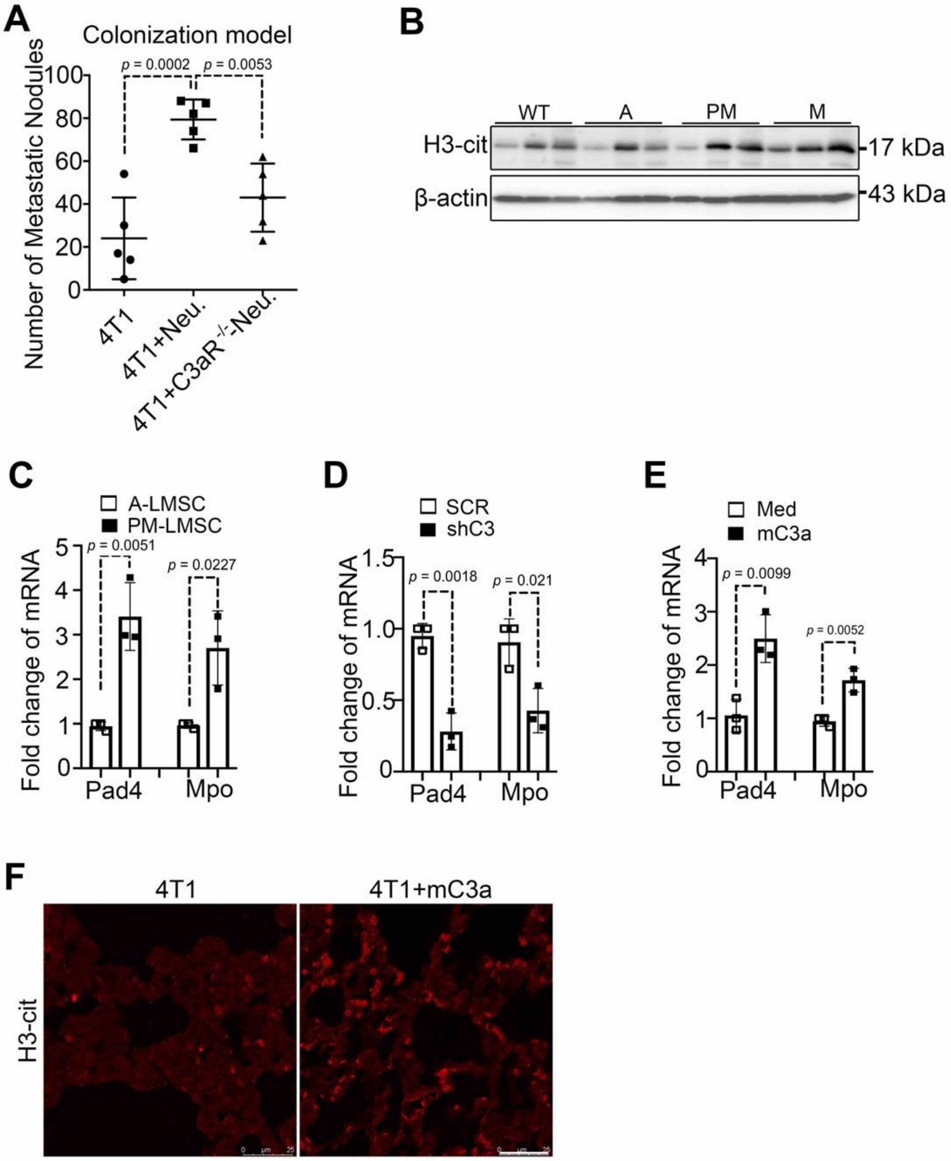

A
Colonization model
Number of Metastatic Nodules
p = 0.0002
p = 0.0053
100
80
60
40
20
0
4T1
4T1+Neu.
4T1+C3aR-/--Neu.
B
WT
A
PM
M
H3-cit
17 kDa
β-actin
43 kDa
C
A-LMSC
PM-LMSC
p = 0.0051
p = 0.0227
Fold change of mRNA
Pad4
Mpo
D
SCR
shC3
p = 0.0018
p = 0.021
Fold change of mRNA
Pad4
Mpo
E
Med
mC3a
p = 0.0099
p = 0.0052
Fold change of mRNA
Pad4
Mpo
F
4T1
4T1+mC3a
H3-cit

Supplementary Figure 4. NET formation is positively regulated by the C3-C3aR axis, related to Fig. 4.

(A) Adoptively transferred *C3aR*$^{-/-}$neutrophils failed to promote metastasis. Neutrophils (4×10^6) from WT and *C3aR*$^{-/-}$ mice were co-injected with 4T1 cells (5×10^5) into BALB/c mice. Lung metastatic nodules were counted on day 14. $n = 5$ mice each.

(B) Proteins from lung tissues of MMTV-PyMT mice at different tumor stages were examined for H3-cit by western blotting analysis. The images were representative of two independent experiments.

(C) PM-LMSCs upregulated NETs associated genes in neutrophils. Neutrophils (1×10^6) were co-cultured with A-LMSCs or PM-LMSCs for 24 hr in the transwell system and were examined for Pad4 and Mpo genes. n = 3 independent experiments.

(D) Knockdown of C3 in LMSCs results in reduced expression of NETs associated genes in neutrophils. Neutrophils (1×10^6) were co-cultured with SCR or shC3 PM-LMSCs for 24 hr in the transwell system and the expression of Pad4 and Mpo genes was determined by qRT-PCR. n = 3 independent experiments.

(E) mC3a induced neutrophils to express NETs associated genes. Neutrophils (1×10^6) were treated with mC3a (1 μg/ml) for 24 hr and examined for the expression of Pad4 and Mpo genes. n = 3 independent experiments.

(F) Immunofluorescence of H3-cit. H3-cit (red) signals were increased in lung tissues in mC3a-treated mice in comparison to control. The scale bar represents 25 μm. The images were representative of those generated from three mice each group.

All the data are presented as mean values +/- SD. ns, not significant; $p < 0.05$, significant, using a one-way ANOVA with Sidak post-test for Supplementary Fig. 4A; using an unpaired, two-tailed, Student's t-test for Supplementary Fig. 4C-E. Source data are provided as a Source Data file for Supplementary Fig. 4A-E.

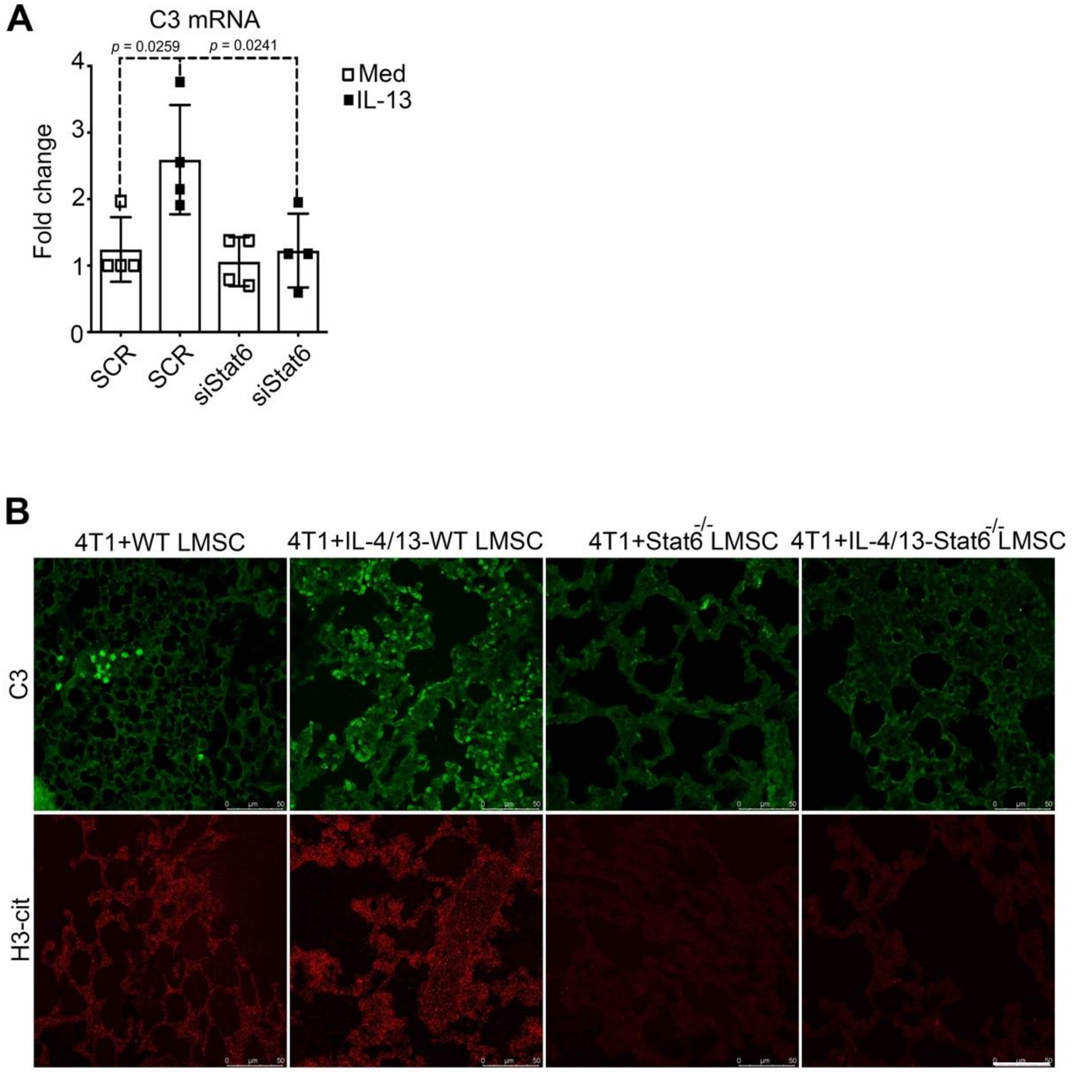
A
C3 mRNA
p = 0.0259
p = 0.0241
Med
IL-13
Fold change
0
1
2
3
4
SCR
SCR
siStat6
siStat6
B
4T1+WT LMSC
4T1+IL-4/13-WT LMSC
4T1+Stat6-/- LMSC
4T1+IL-4/13-Stat6-/- LMSC
C3
H3-cit

Supplementary Figure 5. STAT6 is important for C3 upregulation in PM-LMSCs, related to Fig. 5.

(A) Knockdown of Stat6 in LMSCs abolished IL-13-induced C3 expression. LMSCs were treated with Stat6 siRNA for 24 hr and were then treated with IL-13 for another 24 hr. C3 mRNA was measured by qRT-PCR. $n = 4$ independent cell samples. Data are presented as mean values +/- SD. $p < 0.05$, significant, using a one-way ANOVA with Sidak post-test. Source data are provided as a Source Data file.

(B) Immunofluorescence of C3 and H3-cit. C3 (green) and H3-cit (red) signals were increased in lung tissues of WT LMSCs-treated mice when stimulated with IL-4/13, but not in those treated with $Stat6^{-/-}$ LMSCs. The scale bar represents 50 μm. The images were representative of those generated from three mice each group.

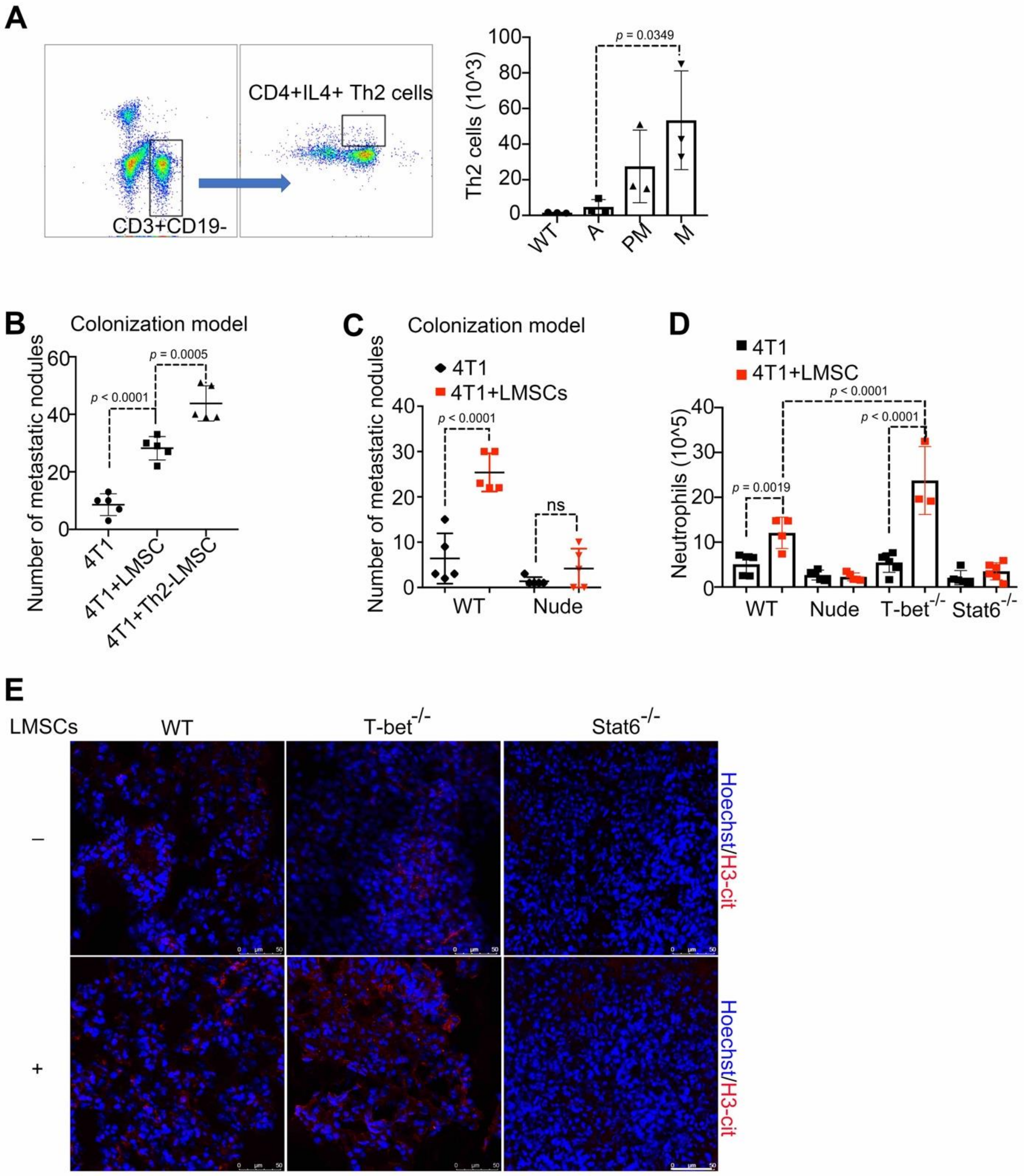

A
CD4+IL4+ Th2 cells
CD3+CD19-
p = 0.0349
Th2 cells (10^3)
WT
A
PM
M
B
Colonization model
p < 0.0001
p = 0.0005
Number of metastatic nodules
4T1
4T1+LMSC
4T1+Th2-LMSC
C
Colonization model
4T1
4T1+LMSCs
p < 0.0001
ns
Number of metastatic nodules
WT
Nude
D
4T1
4T1+LMSC
p < 0.0001
p < 0.0001
p = 0.0019
Neutrophils (10^5)
WT
Nude
T-bet-/-
Stat6-/-
E
LMSCs
WT
T-bet-/-
Stat6-/-
–
+
Hoechst/H3-cit
Hoechst/H3-cit

Supplementary Figure 6. Th2 cells promote lung metastasis, related to Fig. 6

(A) The populations of $CD4^+$ T cells and Th2 cells were increasing during tumor progression. Single-cell suspensions prepared from the lung tissues of MMTV-PyMT mice at different tumor stages were analyzed for the numbers of $CD4^+$ T cells and $CD4^+IL\text{-}4^+$ Th2 cells. $n = 3$ mice each.

(B) Th2 culture medium (Th2-CM)-treated LMSCs acquired enhanced ability to promote lung metastasis. 4T1 cells were co-administered with LMSCs with or without Th2-CM treatment for 24 hr at a 5:1 ratio (5×10^4 4T1 cells and 1×10^4 LMSCs) into BALB/c mice by intravenous injection. After 14 days, lung metastatic nodules were counted. $n = 5$ mice each.

(C) T cells are required for sustaining the metastasis-promoting activity of LMSCs. 4T1 cells and LMSCs were intravenously co-injected into BALB/c or nude mice at a 5:1 ratio (5×10^4 4T1 cells and 1×10^4 LMSCs). After 14 days, lung metastatic nodules were counted. $n = 5$ mice each.

(D) Neutrophil accumulation in the lung requires Th2 cells. LMSCs were co-injected with 4T1 cells at a 1:5 ratio (1×10^4 LMSCs and 5×10^4 4T1 cells) into BALB/c, Nude, *T-bet*$^{-/-}$, *Stat6*$^{-/-}$ mice, and the population of neutrophils was analyzed on day 14. n = 5, 4, 5, 5, 6, 3, 5, 6 mice.

(E) Immunofluorescence of H3-cit. LMSCs or PBS was injected into wild type, T-bet$^{-/-}$ or Stat6$^{-/-}$ mice respectively. The mice were euthanized for the examination of NETs in the lungs 24 hr after injection of LMSCs. n = 3 mice in each group. Scale bar represents 50 µm.

All the data are presented as mean values +/- SD. ns, not significant; $p < 0.05$, significant, using a one-way ANOVA with Sidak post-test. Source data are provided as a Source Data file for Supplementary Fig. 6A-D.

A

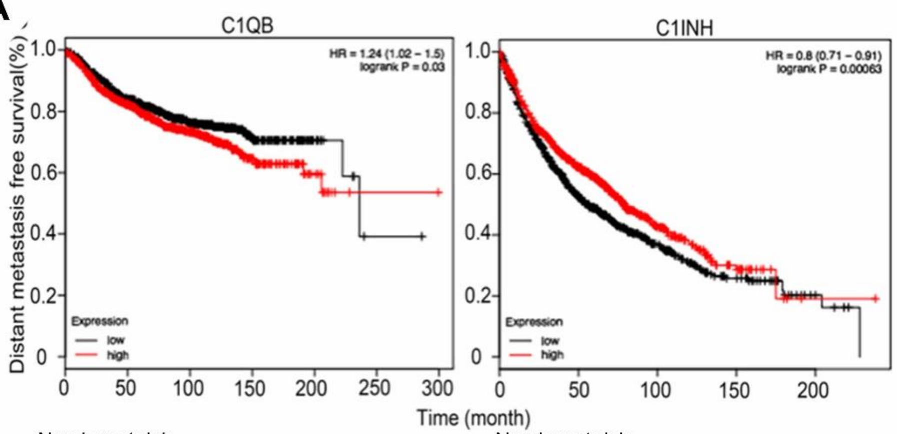

Number at risk

high	873	601	262	73	13	1	0
low	873	639	330	92	14	1	0

Number at risk

high	963	445	88	21	1
low	963	383	115	36	6

Supplementary Figure 7. Complement component is associated with metastasis in breast cancer patients, related to Fig. 7.

(A) Analysis of the database of potential breast cancer biomarkers, high-C1QB and low-C1INH were correlated with poor distant metastasis-free survival.

Supplementary Table 1. Information of clinical tissue samples.

Supplementary Table 1. patient lung tissue sections			
Sample Number	Gender	Sample character	Metastasis (Yes or No)
1#	Female	Lung tissue section/Breast cancer	Y
2#	Female	Lung tissue section/Breast cancer	Y
3#	Female	Lung tissue section/Breast cancer	Y
4#	Female	Lung tissue section/Breast cancer	Y
5#	Female	Lung tissue section/Breast cancer	Y
6#	Female	Lung tissue section/Breast cancer	Y
7#	Female	Lung tissue section/Breast cancer	Y
8#	Female	Lung tissue section/Breast cancer	Y
9#	Female	Lung tissue section/Breast cancer	Y

Supplementary Table 2. Information of clinical serum samples.

Supplementary Table 2. Clinical serum samples			
Sample Number	Gender	Sample character	Metastasis (Yes or No)
1#	Female	Serum/Breast cancer	Y
2#	Female	Serum/Breast cancer	Y
3#	Female	Serum/Breast cancer	Y
4#	Female	Serum/Breast cancer	Y
5#	Female	Serum/Breast cancer	Y
6#	Female	Serum/Breast cancer	Y
7#	Female	Serum/Breast cancer	Y
8#	Female	Serum/Breast cancer	Y
9#	Female	Serum/Breast cancer	N
10#	Female	Serum/Breast cancer	N
11#	Female	Serum/Breast cancer	N
12#	Female	Serum/Breast cancer	N
13#	Female	Serum/Breast cancer	N
14#	Female	Serum/Breast cancer	N
15#	Female	Serum/Breast cancer	N
16#	Female	Serum/Breast cancer	N
17#	Female	Serum/Breast cancer	N
18#	Female	Serum/Breast cancer	N
19#	Female	Serum/Breast cancer	N
20#	Female	Serum/Breast cancer	N
21#	Female	Serum/Breast cancer	N
22#	Female	Serum/Breast cancer	Y
23#	Female	Serum/Breast cancer	Y
24#	Female	Serum/Breast cancer	Y
25#	Female	Serum/Breast cancer	Y
26#	Female	Serum/Breast cancer	Y
27#	Female	Serum/Breast cancer	Y
28#	Female	Serum/Breast cancer	Y

29#	Female	Serum/Breast cancer	Y
30#	Female	Serum/Breast cancer	Y
31#	Female	Serum/Breast cancer	Y
32#	Female	Serum/Breast cancer	N
33#	Female	Serum/Breast cancer	N
34#	Female	Serum/Breast cancer	N
35#	Female	Serum/Breast cancer	N
36#	Female	Serum/Breast cancer	N
37#	Female	Serum/Breast cancer	N
38#	Female	Serum/Breast cancer	N
39#	Female	Serum/Breast cancer	N
40#	Female	Serum/Breast cancer	N
41#	Female	Serum/Breast cancer	N
42#	Female	Serum/Health	N/A
43#	Female	Serum/Health	N/A
44#	Female	Serum/Health	N/A
45#	Female	Serum/Health	N/A
46#	Female	Serum/Health	N/A
47#	Female	Serum/Health	N/A
48#	Female	Serum/Health	N/A

Supplementary Table 3. Information of Oligonucleotides

Oligonucleotides (Murine)		
Primer: *β-actin* (Fw): 5’-CCACGAGCGGTTCCGATG-3’	This paper	N/A
Primer: *β-actin* (Rev): 5’-GCCACAGGATTCCATACCCA-3’	This paper	N/A
Primer: *C3* (Fw): 5’-GAGCGAAGAGACCATCGTACT-3’	PrimerBank	ID: 126518316c1
Primer: *C3* (Rev): 5’-TCTTTAGGAAGTCTTGCACAGTG-3’	PrimerBank	ID: 126518316c1
Primer: *Krt18* (Fw): 5’-CAGCCAGCGTCTATGCAGG-3’	PrimerBank	ID: 254540067c1
Primer: *Krt18* (Rev): 5’-CCTTCTCGGTCTGGATTCCAC-3’	PrimerBank	ID: 254540067c1
Primer: *Krt8* (Fw): 5’-TCCATCAGGGTGACTCAGAAA-3’	PrimerBank	ID: 114145560c1
Primer: *Krt8* (Rev): 5’-AAGGGGCTCAACAGGCTCT-3’	PrimerBank	ID: 114145560c1
Primer: *Spint2* (Fw): 5’-TCCCTCGCTGGTACTATGACA-3’	PrimerBank	ID: 127139426c1
Primer: Spint2 (Rev): 5’-GGGAGAGGTAACTGTTCTTGTTG-3’	PrimerBank	ID: 127139426c1
Primer: *Mpo* (Fw): 5’-AGGGCCGCTGATTATCTACAT-3’	PrimerBank	ID: 226823249c1
Primer: *Mpo* (Rev): 5’-CTCACGTCCTGATAGGCACA-3’	PrimerBank	ID: 226823249c1
Primer: *Padi4* (Fw): 5’-GACCACAACAGTTCTCGTATTGC-3’	PrimerBank	ID: 156938251c2
Primer: *Padi4* (Rev): 5’-CGGGTTAGACTTGTCCAGCAG-3’	PrimerBank	ID: 156938251c2
shRNA: C3: 5’-CCAGAAACAGUGCGAAGAA-3’	Genepharma	N/A
shRNA: Control: 5’-TTCTCCGAACGTGTCACGT-3’	Genepharma	N/A
siRNA: Stat6: 5’-GUGAAAGCCUGGUGGAAAU-3’ (sense)	Genepharma	N/A
siRNA: Control: 5’-UUCUCCGAACGUGUCACGUTT-3’ (sense)	Genepharma	N/A
