# Supplementary material for: Lung mesenchymal stromal cells influenced by Th2 cytokines mobilize neutrophils and facilitate metastasis by producing complement C3
Source: Nat Commun. 2021 Oct 27;12:6202. doi: 10.1038/s41467-021-26460-z (PMC8551331; doi:10.1038/s41467-021-26460-z)
Supplement: Supplementary file 2 — Reporting Summary [file 41467_2021_26460_MOESM2_ESM.pdf]

nature research

Corresponding author(s): Yufang Shi

Last updated by author(s): Sep 22, 2021

Reporting Summary

Nature Research wishes to improve the reproducibility of the work that we publish. This form provides structure for consistency and transparency in reporting. For further information on Nature Research policies, see our Editorial Policies and the Editorial Policy Checklist.

Statistics

For all statistical analyses, confirm that the following items are present in the figure legend, table legend, main text, or Methods section.

n/a	Confirmed	
☐	☒	The exact sample size (*n*) for each experimental group/condition, given as a discrete number and unit of measurement
☐	☒	A statement on whether measurements were taken from distinct samples or whether the same sample was measured repeatedly
☐	☒	The statistical test(s) used AND whether they are one- or two-sided<br>*Only common tests should be described solely by name; describe more complex techniques in the Methods section.*
☒	☐	A description of all covariates tested
☐	☒	A description of any assumptions or corrections, such as tests of normality and adjustment for multiple comparisons
☐	☒	A full description of the statistical parameters including central tendency (e.g. means) or other basic estimates (e.g. regression coefficient) AND variation (e.g. standard deviation) or associated estimates of uncertainty (e.g. confidence intervals)
☐	☒	For null hypothesis testing, the test statistic (e.g. *F*, *t*, *r*) with confidence intervals, effect sizes, degrees of freedom and *P* value noted<br>*Give P values as exact values whenever suitable.*
☒	☐	For Bayesian analysis, information on the choice of priors and Markov chain Monte Carlo settings
☒	☐	For hierarchical and complex designs, identification of the appropriate level for tests and full reporting of outcomes
☒	☐	Estimates of effect sizes (e.g. Cohen's *d*, Pearson's *r*), indicating how they were calculated

Our web collection on statistics for biologists contains articles on many of the points above.

Software and code

Policy information about availability of computer code

Data collection	RNA-seq were collected using illumina Hiseq 2000/2500. FACS data were acquired on a Beckman CytoFlex.
Data analysis	GrapPhpad Prism (version 8), FlowJo (X 10.0.7r2), CytExpert (2.3.0.28), limma (3.46.0), pheatmap (1.0.12)

For manuscripts utilizing custom algorithms or software that are central to the research but not yet described in published literature, software must be made available to editors and reviewers. We strongly encourage code deposition in a community repository (e.g. GitHub). See the Nature Research guidelines for submitting code & software for further information.

Data

Policy information about availability of data

All manuscripts must include a data availability statement. This statement should provide the following information, where applicable:

- Accession codes, unique identifiers, or web links for publicly available datasets
- A list of figures that have associated raw data
- A description of any restrictions on data availability

LMSCs sequencing data have been deposited in the GEO under accession GSE125591 and GSE179315. Figure 2A and Figure S2A have the associated raw data.

Field-specific reporting

Life sciences study design

All studies must disclose on these points even when the disclosure is negative.

Sample size	Sample sizes were determined based on our experience with the specific type of experiment and commonly used sample sizes in comparable publications within this field of research. For an example, the sizes of different groups of MMTV-pyMT mice were not the same at the same time, but it is confirmed that n ≥ 3. The sample sizes and number of repeats are defined in each figure legends.
Data exclusions	Data were not excluded unless the mice were dead before analysis.
Replication	Experimental findings were reliably reproduced within our lab, based on at least 3 biological replicates for each experiment (Figure S3C and S3E were not included).
Randomization	All animal experiments were grouped randomly. Experimental groups were formed based on genotypes and genetically related cohorts, when possible.
Blinding	For in vivo studies, investigators were blinded for data acquisition and data analysis.

Reporting for specific materials, systems and methods

We require information from authors about some types of materials, experimental systems and methods used in many studies. Here, indicate whether each material, system or method listed is relevant to your study. If you are not sure if a list item applies to your research, read the appropriate section before selecting a response.

Materials & experimental systems

n/a	Involved in the study	
☐	☒	Antibodies
☐	☒	Eukaryotic cell lines
☒	☐	Palaeontology and archaeology
☐	☒	Animals and other organisms
☐	☒	Human research participants
☒	☐	Clinical data
☒	☐	Dual use research of concern

Methods

n/a	Involved in the study	
☒	☐	ChIP-seq
☐	☒	Flow cytometry
☒	☐	MRI-based neuroimaging

Antibodies

Antibodies used

Armenian hamster monoclonal anti-CD29, PE-conjugated (clone HMb1-1) eBioscience Cat#12-0291-81;
Rat monoclonal anti-CD44, FITC-conjugated (clone IM7) eBioscience Cat#11-0441-85;
Rat monoclonal anti-CD44, PE-conjugated (clone IM7) eBioscience Cat#12-0441-83;
Rat monoclonal anti-mouse Ly-6A/E (Sca-1), PE-Cy7-conjugated (clone D7) BioLegend Cat#108114;
Rat monoclonal anti-mouse CD140a, PE-conjugated (clone APA5) eBioscience Cat#12-1401-81;
Rat anti-mouse Hematopoietic Lineage Cocktail, eFlour 450-conjugated eBioscience Cat#88-7772-72
7-amino-actinomycin D (7AAD) Viability Staining Solution eBioscience Cat#00-6993-50
Rabbit monoclonal anti-mouse CD45, PE-conjugated (clone I3/2.3) BioLegend Cat#147712;
Rat monoclonal anti-mouse F4/80, APC-conjugated (clone BM8) eBioscience Cat#17-4801-82;
Rat monoclonal anti-mouse CD11b, FITC-conjugated (clone M1/70) eBioscience Cat#11-0112-85;
Rat monoclonal anti-mouse Ly-6G/Ly6C, PE-conjugated (clone RB6-8C5) eBioscience Cat#12-5931-83;
Rat monoclonal anti-mouse CD8a, APC-conjugated (clone 53-6.7) BioLegend Cat#100712
Rat anti-mouse CD4, APC-H7-conjugated (clone GK1.5) BD Bioscience Cat#560181
Rat anti-mouse CD4, FITC-conjugated (clone GK1.5) eBioscience Cat#11-0041-85
Rat monoclonal anti-mouse CD3, Pacific Blue-conjugated (clone 17A2) BioLegend Cat#100214;
Rat anti-mouse CD19, BV510-conjugated (clone 1D3) BD Bioscience Cat#562956
Rat anti-mouse CD19, BV605-conjugated (clone 6D5) BioLegend Cat#115540
Rat monoclonal anti-mouse IL4, PE-conjugated (clone 11B11) eBioscience Cat#12-7041-82;
Rat monoclonal anti-mouse IFN gamma, FITC-conjugated (clone XMG1.2) eBioscience Cat#11-7311-81;
Goat polyclonal Secondary Antibody to Rat IgG-H&L (Alexa Fluor 488) Abcam Cat#ab150157;
Goat polyclonal Secondary Antibody to Rabbit IgG-H&L (Alexa Fluor 488) Abcam Cat#ab150077;
Goat polyclonal Secondary Antibody to Rat IgG-H&L (Alexa Fluor 594) Abcam Cat#ab150160;
Donkey Secondary Antibody to Rabbit IgG-H&L (Alexa Fluor 555) Beyotime Cat#A0453
Goat Secondary Antibody to Mouse IgG-H&L (Alexa Fluor 47) Beyotime Cat#A0473
Rabbit polyclonal to Histone H3 antibody (citrulline R2 + R8 + R17)-ChIP Grade Abcam Cat#ab5103;
Rat monoclonal anti-mouse C3 (clone 11H9) Abcam Cat#ab11862;
Rabbit monoclonal anti-C3 (clone EPR19394) Abcam Cat#ab200999
Rat monoclonal anti-mouse Ly6g (clone RB6-8C5) Abcam Cat#ab25377;

	Rabbit monoclonal anti-Myeloperoxidase (clone EPR20257) Abcam Cat#ab208670<br>Rat monoclonal anti-mouse Nestin (clone 7A3) Abcam Cat#ab81462;<br>Mouse monoclonal anti-mouse β-Actin (clone AC-15) Sigma Cat#A5441;<br>Mouse monoclonal anti-human Nestin (clone 10C2) Abcam Cat#ab22035;<br>Rabbit polyclonal anti-human C3 Abcam Cat#ab97462;<br>Rabbit monoclonal anti-Phospho-Stat6 (Tyr641) (clone D8S9Y) Cell Signaling TECHNOLOGY Cat#56554<br>Ultra-LEAF™ Purified anti-mouse Ly-6G BioLegend Cat#127649<br>LEAF™ Purified Rat IgG2a, κ Isotype Ctrl BioLegend Cat#400544
Validation	All antibodies for the flow cytometry analyses were obtained from commercial retailers. They were all validated by the manufacturers and data are available on their website.

Eukaryotic cell lines

Policy information about cell lines

Cell line source(s)	4T1 cells (ATCC, CRL-2539) were purchased Stem Cell Bank, Chinese Academy of Sciences.
Authentication	The 4T1 cells were not authenticated upon arrival.
Mycoplasma contamination	All cell lines tested negative for mycoplasma contamination.
Commonly misidentified lines (See ICLAC register)	No cell lines used in this study were found in the database of commonly misidentified cell lines that is maintained by ICLAC and NCBI Biosample.

Animals and other organisms

Policy information about studies involving animals; ARRIVE guidelines recommended for reporting animal research

Laboratory animals	Nude (nu/nu) Jackson Laboratory NU/J, female, 6-8 week old.<br>MMTV-PyMT Jackson Laboratory FVB/N-Tg (MMTV-PyVT) 634Mul/J, female, 7-13 week old.<br>Stat6-/- Jackson Laboratory C.129S2-Stat6tm1Gru/J, female, 6-8 week old.<br>T-bet-/- Jackson Laboratory C.129S6-Tbx21tm1Glm/J, female, 6-8 week old.<br>C3-/- Jackson Laboratory B6.129S4-C3tm1Crr/J, female, 6-8 week old.<br>C3aR-/- Jackson Laboratory C.129S4-C3ar1tm1Cge/J, female, 6-8 week old.<br>BALB/c Jackson Laboratory BALB/c J, female, 6-8 week old.<br>FVB Jackson Laboratory FVB/NJ, female, 6-8 week old.
Wild animals	No wild animals were used here.
Field-collected samples	The study did not involve samples collected from the field.
Ethics oversight	Mice were obtained from the Jackson Laboratory and bred in a specific pathogen-free animal facility of Soochow University. The animal protocols for the experiments described in this paper were approved by the Ethical Committee of Soochow University.

Note that full information on the approval of the study protocol must also be provided in the manuscript.

Human research participants

Policy information about studies involving human research participants

Population characteristics	All the characteristics of the human research participants are listed in Table S1 and Table S2.
Recruitment	Samples were obtained from the First Affiliated Hospital of Soochow University. The participants were voluntary. There is no self-selection bias.
Ethics oversight	All tissues and sera were obtained with informed consent compliance with the Ethical Committee of the First Affiliated Hospital of Soochow University.

Note that full information on the approval of the study protocol must also be provided in the manuscript.

Flow Cytometry

Plots

Confirm that:

- [x] The axis labels state the marker and fluorochrome used (e.g. CD4-FITC).
- [x] The axis scales are clearly visible. Include numbers along axes only for bottom left plot of group (a 'group' is an analysis of identical markers).
- [x] All plots are contour plots with outliers or pseudocolor plots.
- [x] A numerical value for number of cells or percentage (with statistics) is provided.

Methodology

Sample preparation	Characterization of the surface markers in the various cell types in different tissues were performed by flow cytometry. Lung tissue was digested using type II collagenase (Thermo Fisher 17101015) in Dulbecco's modification of Eagle's medium for 1h at 37 ℃, and the digested samples were then filtered through a 70 µm cell strainer, washed and resuspended in PBS supplemented with 2% bovine serum albumin (FBS). For the MMTV-pyMT mice, the resuspended volume of PM and M groups were reduced to 1/3 to obtain the similar cells to stain. Total cell counts were determined using flow cytometry. For surface marker analysis, cells isolated from lung tissues were suspended in staining buffer (PBS, 5% FBS) at a concentration of 1000000 cells/ml and 50 µL of suspension was incubated with fluorescently labeled antibodies at 96-well plate for 30 min at 4 ℃. After washing, the cells were resuspended for analysis or further stained for intracellular analysis.
Instrument	Cytoflex, Beckman Coulter
Software	Flow Jo v10 and Cytexpert software
Cell population abundance	The purity of sorted cell populations was more than 95%, as verified by flow cytometry.
Gating strategy	For all experiments, cells were first gated SSC-H and FSC-H to obtain the cells. Then, target cell population for further analysis were gated by cell surface marker. Please see the FACS gating strategies in Supplementary Figure 1A.

- [x] Tick this box to confirm that a figure exemplifying the gating strategy is provided in the Supplementary Information.
